# The effects of an 8-week mindful eating intervention on anticipatory reward responses in striatum and midbrain

**DOI:** 10.3389/fnut.2023.1115727

**Published:** 2023-08-11

**Authors:** Lieneke K. Janssen, Iris Duif, Anne E. M. Speckens, Ilke van Loon, Joost Wegman, Jeanne H. M. de Vries, Roshan Cools, Esther Aarts

**Affiliations:** ^1^Donders Institute for Brain, Cognition and Behavior, Radboud University, Nijmegen, Netherlands; ^2^Department of Neurology, Max Planck Institute for Human Cognitive and Brain Sciences, Leipzig, Germany; ^3^Department of Psychiatry, Radboud University Medical Center, Nijmegen, Netherlands; ^4^Division of Human Nutrition and Health, Wageningen University, Wageningen, Netherlands

**Keywords:** reward anticipation, mindful eating, striatum, midbrain, fMRI

## Abstract

**Introduction:**

Accumulating evidence suggests that increased neural responses during the anticipation of high-calorie food play an important role in the tendency to overeat. A promising method for counteracting enhanced food anticipation in overeating might be mindfulness-based interventions (MBIs). However, the neural mechanisms by which MBIs can affect food reward anticipation are unclear. In this randomized, actively controlled study, the primary objective was to investigate the effect of an 8-week mindful eating intervention on reward anticipation. We hypothesized that mindful eating would decrease striatal reward anticipation responses. Additionally, responses in the midbrain—from which the reward pathways originate—were explored.

**Methods:**

Using functional magnetic resonance imaging (fMRI), we tested 58 healthy participants with a wide body mass index range (BMI: 19–35 kg/m^2^), motivated to change their eating behavior. During scanning they performed an incentive delay task, measuring neural reward anticipation responses to caloric and monetary cues before and after 8 weeks of mindful eating or educational cooking (active control).

**Results:**

Compared with the educational cooking intervention, mindful eating affected neural reward anticipation responses, with reduced caloric relative to monetary reward responses. This effect was, however, not seen in the striatum, but only in the midbrain. The secondary objective was to assess temporary and long-lasting (1 year follow-up) intervention effects on self-reported eating behavior and anthropometric measures [BMI, waist circumference, waist-to-hip-ratio (WHR)]. We did not observe effects of the mindful eating intervention on eating behavior. Instead, the control intervention showed temporary beneficial effects on BMI, waist circumference, and diet quality, but not on WHR or self-reported eating behavior, as well as long-lasting increases in knowledge about healthy eating.

**Discussion:**

These results suggest that an 8-week mindful eating intervention may have decreased the relative salience of food cues by affecting midbrain but not striatal reward responses, without necessarily affecting regular eating behavior. However, these exploratory results should be verified in confirmatory research.

The primary and secondary objectives of the study were registered in the Dutch Trial Register (NTR): NL4923 (NTR5025).

## 1. Introduction

Reward-related disorders such as addiction, binge-eating disorder and obesity, are characterized by altered responses to reward cues related to the target of abuse (1–3). Mesolimbic regions in the brain, including the striatum and the midbrain—with its dopaminergic projections to the striatum (4, 5)—respond to increases in appetitive motivation induced by reward cues (6). Responses of these subcortical reward regions have been related to eating behavior. For example, greater ventral striatal responses to reward cues have been associated with subsequent food intake (7) and future weight gain in healthy- and overweight individuals (7–9) [for a review, see (3)]. Reductions in striatal food-cue responses after a weight loss intervention were even predictive of the later outcome of the weight loss intervention (10). Moreover, increases in BMI—from lean to obese—were associated with increased midbrain responses to high-calorie food cues in women (11) and to anticipating rewards during risky choices in adolescents (12). Interventions targeted at diminishing subcortical responses to food reward cues may therefore be promising for addressing people’s tendency to overeat.

Mindfulness-based interventions are aimed at cultivating attention to present-moment experience, without judgment (13). Protocolized mindfulness interventions, such as mindfulness-based stress reduction (MBSR) have shown to be effective in reducing subcortical responses to emotional stimuli in anxiety (14) as well as in healthy individuals (15). Furthermore, mindfulness meditation training can improve executive control processes such as conflict monitoring and response inhibition (16), as well as alter functional connectivity of brain networks involved in attention, cognitive processing, awareness, sensory integration, and reward processing (17). Importantly, mindfulness-based interventions aimed at changing eating behavior have been shown to reduce binge eating in clinical populations (18, 19), to reduce abdominal fat (20, 21), to increase self-reported mindful eating (22) and to reduce reward-driven eating in obese individuals (23). However, only two of these trials were actively controlled (18, 22, 23). It is therefore unclear whether these beneficial effects can be attributed to mindfulness *per se*. In fact, Kristeller et al. (18) found that both mindfulness-based eating awareness training (MB-EAT) and a psycho-educational/cognitive-behavioral (i.e., active control) intervention decreased binge-eating symptoms relative to a waitlist control group to a similar degree. Given the different nature of these interventions, it is possible that reduced symptomatology was mediated by dissociable brain mechanisms, as has previously been observed in an actively controlled clinical trial on social anxiety (14). In this fMRI study, reduced social anxiety symptoms were observed for both the mindfulness and the active control intervention, but the interventions had opposite effects on neural responses in the posterior cingulate and the dorsomedial prefrontal cortex during self-referential processing Studies investigating the neurocognitive mechanism underlying mindful eating are required to assess whether a mindful eating intervention can diminish neural responses to food reward cues.

Kirk et al. (24) performed three studies on neurocognitive reward mechanisms underlying mindfulness. They found that meditators, relative to controls, showed lower neural responses in striatum during reward anticipation (24), as well as diminished BOLD responses in putamen during positive and negative prediction errors (25). In addition, they found that mindfulness training modulated value signals in vmPFC to primary reward (juice) delivery (26). However, these studies do not yet address the question how mindfulness training affects neural responses for food reward anticipation. Specifically, the first two studies were performed in experienced meditators vs. controls instead of in a randomized controlled design, and the third study investigated reward responses at the moment of reward delivery, instead of anticipation. Reward anticipation is particularly interesting to investigate in light of eating behavior, as increases in reward anticipation have been shown to have predictive value for weight gain or overeating-related behavior (1–3, 7–9, 12).

Here, we present an actively controlled randomized study investigating the effects of mindfulness on reward anticipation in the brain of healthy adults who wanted to change their eating behavior for various reasons. We studied the effects of an 8-week mindful eating intervention aimed at changing undesired eating habits vs. a carefully matched educational cooking intervention (active control). To assess reward anticipation, we used an incentive delay task (27) during fMRI, which has been shown to produce reliable mesolimbic responses to reward cues (5). We hypothesized that the mindful eating intervention would reduce reward cue responses in the striatum (primary objective), and also explored these effects in the dopaminergic midbrain as part of the mesolimbic reward circuit. We included both monetary and caloric rewards in the task, which enabled us to assess whether the effect on anticipatory reward responses is specific to the caloric domain, or generalizes to the monetary domain. As a secondary objective, we assessed the effects of mindful eating on anthropometric measures (BMI, waist-to-hip ratio (WHR), and waist circumference) and on self-reported questionnaires related to eating behavior and knowledge of healthy eating.

## 2. Materials and methods

### 2.1. Participants

The participants that were included in the current sample (*n* = 58; see Table 1 for group characteristics) largely overlap with those from the sample reported previously (28). Only right-handed adults in the age between 18 and 55 years old, with a BMI between 19 and 35 kg/m^2^, who had an adequate demand of the Dutch language (i.e., who could understand the task instructions, questionnaires, and intervention materials) and who, importantly, were highly motivated to change their eating behavior (not to lose weight *per se*) were included in the study. The exclusion criteria were as follows: MRI-incompatibility; diabetes mellitus; self-reported (history of) hepatic, cardiac, respiratory, renal, cerebro-vascular, endocrine, metabolic, pulmonary, or cardiovascular diseases or of taste or smell impairments; food allergies relevant to the study; uncontrolled hypertension (in rest: diastolic >90 mmHg; systolic >160 mmHg); self-reported (history of) eating, neurological, or psychiatric disorders; a score > 11 on the Hospital Anxiety and Depression Scale (29) (HADS); extremely high restrained eating scores [≥4.00 for women, ≥3.60 for men, based on a normative Dutch sample (30)] on the Dutch Eating Behaviour Questionnaire (31) (DEBQ); self-reported drug or alcohol addiction in the past 6 months; self-reported use of neuroleptic or other psychotropic medication; deafness, blindness, or sensorimotor handicaps; current strict dieting, under treatment with a dietitian, or a change in body weight of more than 5 kg in the past 2 months (self-report). Crucially, subjects with previous MBSR (Mindfulness-Based Stress Reduction) or MBCT (Mindfulness-Based Cognitive Therapy) experience were excluded from the study (self-report).

**Table 1:** Between-group comparisons (ME, mindful eating; EC, educational cooking).

	Mindful eating (ME) (*n* = 32)			Educational cooking (EC) (*n* = 26)			*p*-value	Test-statistic	Effect size^d^
**Minimization factors**									
Gender (male: female)	5: 27			5: 21			0.740	na^a^	na
Age (yrs)	32.3	±10.8	20–52	30.6	±11.3	19–51	0.546	0.607^b^	0.154
Body mass index (kg/m^2^)	26.6	±4.1	19–35	25.5	±3.4	20–33	0.296	1.054^b^	0.292
BMI groups (NW: OW: OB)	11: 14: 7			11: 12: 3			0.648	na^a^	na
Yoga/meditation experience (yrs)	1.0	±2.6	0–14	1.9	±4.3	0–19	0.334	−0.974^b^	0.253
**Sample characterization**									
Education	6.5	±0.6	5–7	6.2	±0.7	5–7	0.053	304.0^c^	−0.033^e^
Digit span (total score)	15.6	±3.5	9–23	14.1	±3.5	9–22	0.120	1.577^b^	0.429
Smoking (FTND score)	0.19	±1.1	0–6	0.04	±0.2	0–1	0.902	413.5^c^	−0.002^e^
**Intervention**									
Time on training (hrs)	31.0	±14.4	2.5–47.8	23.9	±21.2	0–77.7	0.135	1.518^b^	0.392
Attendance <4 sessions (*n*)	5			5			0.740	na^a^	na
Attendance (number of sessions)	6.5	±2.5	1–9	6.3	±2.8	1–9	0.738	0.336^b^	0.075

If not otherwise stated, values denote mean ± SD, and min-max.

NW, normal-weight; OW, overweight; OB, obese; FTND, Fagerstrom Test for Nicotine Dependence.

a Based on Fisher’s Exact Test.

b Independent samples *t*-test (degrees of freedom: 56).

c Mann-Whitney test.

d If not otherwise stated, effect sizes indicate Cohen’s d.

e r, effect size for Mann Whitney U-test [*z*-value divided by the total sample size (58)].

**Figure 1:**
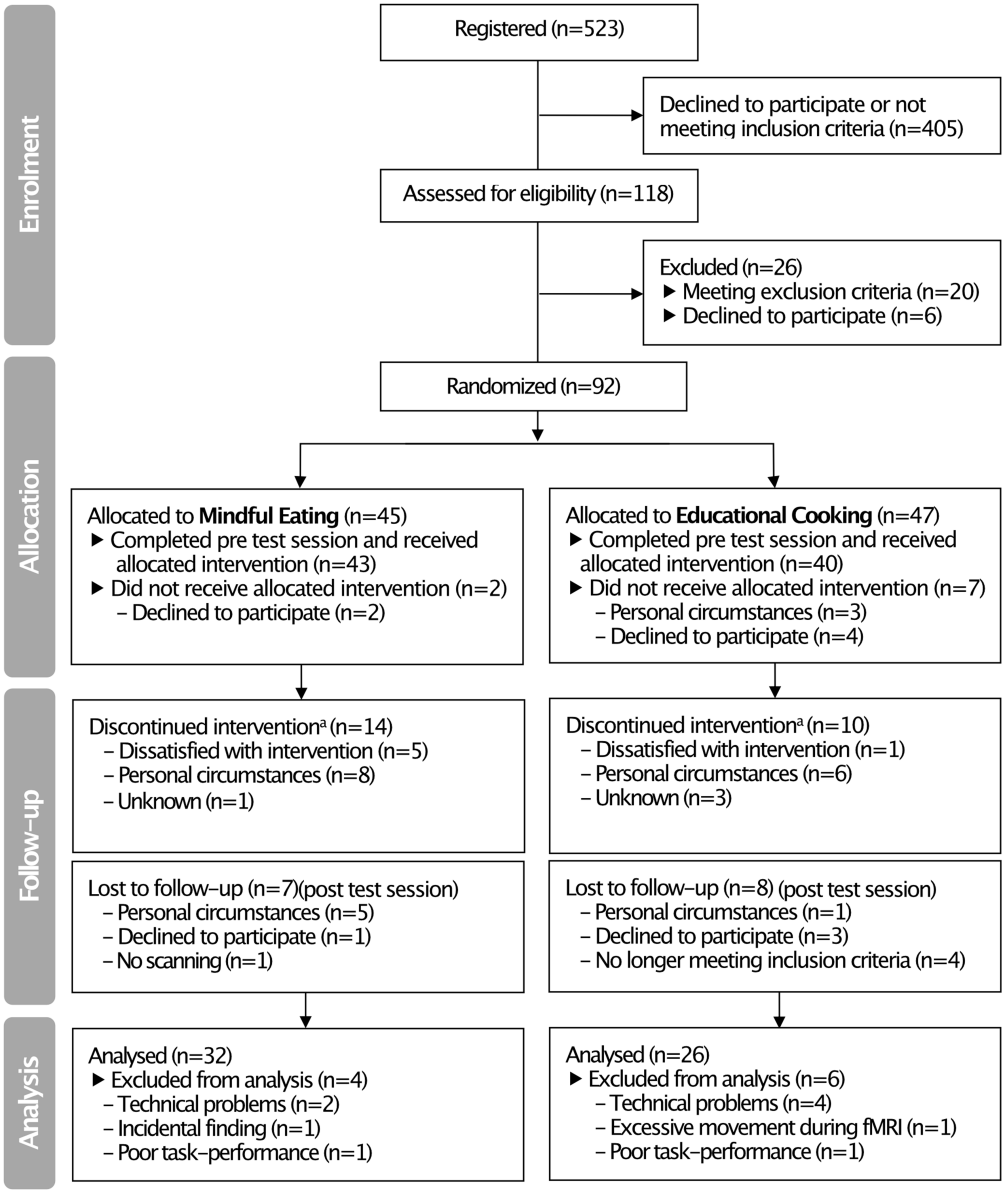
CONSORT flow diagram summarizing the inclusion and exclusion of participants in the pre-intervention (“Allocation”) and immediate post-intervention (“Follow-up”) test sessions. ^a^Attended <4 sessions of the intervention program. Note that these participants were invited back to the laboratory for the *post*-intervention test session.

The study was conducted between October 2013 and February 2016. Participants were recruited from Nijmegen and surroundings through advertisement. From 523 initial registrations (see Figure 1 for a flow diagram), 118 participants were assessed for their eligibility to take part in the study during a separate screening session (Section 2.2). Ninety-two participants were included in the study and randomly allocated to the mindful eating (*n* = 45) or educational cooking (*n* = 47) intervention. Of the included participants, 83 completed a pre-intervention test session and started the allocated intervention (mindful eating: *n* = 43; educational cooking: *n* = 40). Fifteen participants did not return for the post-intervention test session, resulting in a total of 68 complete datasets (mindful eating: *n* = 36; educational cooking: *n* = 32). An additional 10 participants were excluded from the analyses following testing because of technical problems (*n* = 6), excessive movement during fMRI scanning (*n* = 1), an incidental finding after the post-test session (*n* = 1), or because of poor task performance (*n* = 2) (for details see Section 2.6.2). Despite a numerical difference in dropouts between groups, the number of people excluded from analysis was not significantly different [mindful eating: 28.8%, educational cooking: 44.7%, χ^2^(1, *N* = 92) = 2.461, *p* = 0.117]. The relatively high dropout rate is addressed in Section 4. The reasons for discontinuation of the intervention or study were queried by the experimenters and categorized as “dissatisfied,” “personal circumstances” or “unknown” (see Figure 1). Note that the number of included participants was chosen based on typical neuroimaging studies and anticipating dropout due to the interventions. No power calculation was conducted at the time because of a lack of similar studies to extract relevant information from.

All participants who were assessed for their eligibility to take part in the study gave written informed consent and were reimbursed for participation according to the local institutional guidelines (i.e., 8 Euros per hour for behavioral testing, 10 Euros per hour for scanning). The study protocol was approved by the local ethics committee (CMO region Arnhem-Nijmegen, the Netherlands, 2013-188) and was in accordance with the Declaration of Helsinki. The trial was registered at the Dutch trial register [NL4923 (NTR5025)].

### 2.2. Study protocol

In the separate screening interview, all participants were assessed for inclusion and exclusion criteria (listed under Section 2.1) and the matching criteria: age, gender, BMI, and experience with meditation and yoga. Anthropometric measures (weight, height, waist and hip circumference) and physical measures (blood pressure) were collected and a number of self-report questionnaires used for exclusion (listed with the exclusion criteria below) were administered.

After inclusion, participants came to the MRI laboratory twice—before and after the intervention—and a third time to the behavioral lab 1 year later. Participants were instructed to abstain from eating foods and drinking anything else than water 4 h prior to the start of the test sessions. Participants were also instructed to abstain from drinking alcohol 24 h before the test session. As secondary outcome measures, anthropometric measurements were taken again (weight, height, waist and hip circumference) before scanning and participants completed self-reported measures of diet quality and eating behavior: the Dutch Healthy Diet - Food Frequency Questionnaire (32) (DHD-FFQ) on food intake; a shortened version of the Food Behavior Questionnaire (FBQ) with subscales on “knowledge of healthy eating” and “temptation”; and the Dutch Eating Behaviour Questionnaire (31) (DEBQ) with subscales on restraint, emotional, and external eating behaviors. To further characterize the sample, to account for between-group differences at baseline that could occur by chance, and to further explore the effectiveness of the intervention programs, the following self-report questionnaires and scales were administered: the Five Facet Mindfulness Questionnaire – Short Form (33) (FFMQ-SF) to assess degree of mindfulness; a Treatment Credibility Questionnaire (TCQ) to assess how much participants believed the intervention would work for them; the Positive And Negative Affect Scale (34) (PANAS) to assess positive and negative affect before scanning; the Behavioral Inhibition System/Behavioral Approach System questionnaire (35) (BIS-BAS) to assess punishment and reward sensitivity; the Hospital Anxiety and Depression Scale (29) (HADS) to assess levels of anxiety and depression; the Fagerstrom Test for Nicotine Dependence (36) (FTND) to assess smoking and nicotine dependence; the Barratt Impulsiveness Scale-11 (37) (BIS-11) to assess impulsivity; the Kirby monetary choice questionnaire (38) to assess delayed reward discounting; and the neuropsychological digit span test (39) to assess working memory capacity. Note that the pre-training TCQ was filled out at the first training session, not on the pre-training test session, as participants were unaware of the contents of their training at that time. After completing the questionnaires, participants underwent a 1-h MR scanning session in which they performed an incentive delay task, reported here. Participants also performed a food Stroop task inside the scanner, followed by a reversal learning and outcome devaluation task outside the scanner. Data from these three experimental paradigms are reported elsewhere (28, 40, 41).

One year after the intervention, participants were re-invited to the laboratory to reassess anthropometric measurements, i.e., weight, waist and hip circumference, and the self-report questionnaires as administered on pre- and post-test sessions. Reward anticipation was not re-assessed at one-year follow-up.

### 2.3. Interventions

Participants were randomly assigned to one of two intervention programs: mindful eating (ME) or educational cooking (EC; active control). Participants were assigned by a computer through minimization (42), which guarantees that groups are balanced in terms of certain *a priori* determined minimization factors: age (categories: 18–25y, 26–35y, 36–45y, 46–55y), gender (categories: male, female), BMI (categories: 19–24.9 kg/m^2^ normal weight, 25–29.9 kg/m^2^ overweight, 30–35 kg/m^2^ moderately obese) and experience with meditation and yoga (categories: never, 0–2 years, 2–5 years, 5–10 years, >10 years).

The intervention programs were matched in terms of time, effort, and group contact, but differed significantly in terms of content. Both programs consisted of 8 weekly, 2.5 h group sessions plus 1 day (6 h) dedicated to the intervention goals. Participants were asked to spend 45 min per day on homework assignments and to record the amount of time spent on homework forms. In the information letters, the intervention programs were described as “eating with attention” (ME) and “eating with knowledge” (EC) to prevent a selection bias of participants interested in mindfulness. Only after the first test session, participants were informed about the intervention to which they were randomized, to ensure that baseline measurements were not influenced by intervention expectations. Because group size was set to 10–15 participants per round, included participants were divided across three rounds for each intervention (3xME, 3xEC).

#### 2.3.1. Mindful eating

The aim of the ME intervention was to increase experiential awareness of food and eating. The ME program was based on the original MBSR program developed by Kabat-Zinn et al. (43). Participants performed formal mindfulness practices (i.e., body scan, sitting meditation, walking meditation and mindful movement), aimed at increasing general mindfulness skills, which were similar to the original program. In addition, participants performed informal mindfulness practices based on the Mindful Eating, Conscious Living program (44), which were mainly directed to mindful eating and not part of the original MBSR program. Sessions focused on the automatic pilot, perception of hunger and satiation, creating awareness of boundaries in eating behavior, stress-related eating, coping with stress, coping with (negative) thoughts, self-compassion, and how to incorporate mindfulness in daily life. Toward the end of the program, participants had a “silent day.” During this day, the whole group performed formal mindfulness exercises and ate a meal together in complete silence. Homework consisted of a formal mindfulness practice and an informal mindfulness practice directed at one moment (e.g., a meal) a day. The ME intervention was developed and delivered by qualified mindfulness teachers from the Radboud University Medical Centre for Mindfulness.

#### 2.3.2. Educational cooking

The aim of the EC intervention was to increase informational awareness of healthy food and eating. The EC program was based on the Dutch healthy food-based dietary guidelines.^1^ To establish similar (active) group activities as in the ME, participants were enrolled in cooking workshops during the group meetings of the EC. Sessions focused on healthy eating, healthy cooking of vegetables and fruit, use of different types of fat and salt for cooking, reading of nutrition labels on food products, healthy snacking, guidelines for making healthy choices when eating in restaurants, and how to incorporate healthy eating and cooking in daily life. Toward the end of the program, participants had a “balance day,” during which the participants adhered to all nutritional health guidelines for every snack and meal. Homework assignments entailed practicing cooking techniques, or grocery shopping with informational awareness, and counting the amount of calorie intake for one meal a day. The EC intervention was developed and delivered by a qualified dietitian from Wageningen University and a professional chef of the Nutrition and Dietetics faculty of the University of Applied Sciences of Arnhem-Nijmegen guided the cooking sessions.

### 2.4. Incentive delay task

**Figure 2:**
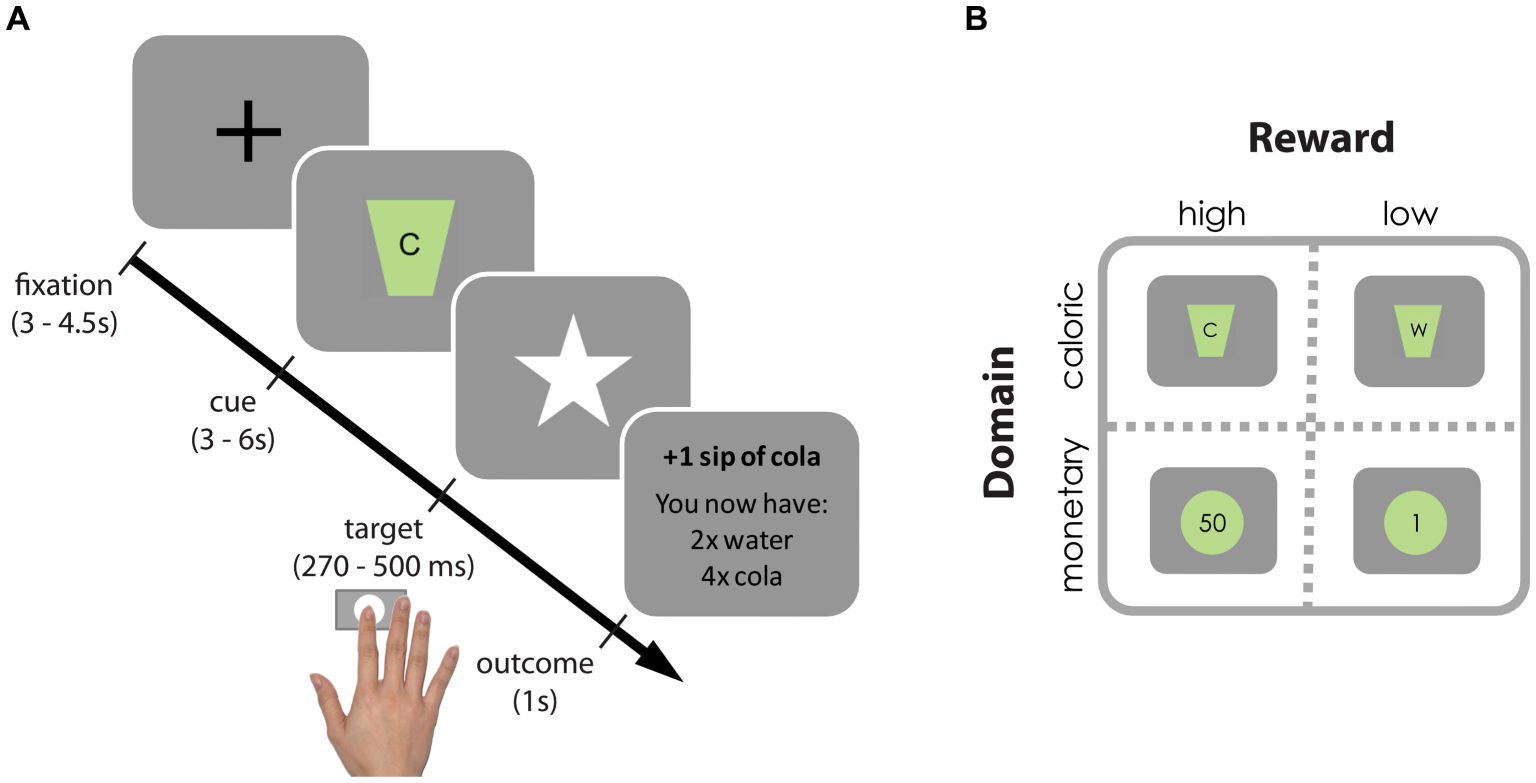
Incentive delay task. **(A)** Each trial started with a fixation cross, followed by a cue signaling which reward could be earned on that trial. Subsequently, a white star (i.e., target) appeared for a brief period and participants were instructed to press a button as fast as possible upon detection using their right index finger. If participants pressed before the response deadline (hit trial), the target remained on the screen, informing participants of the successful registration of their key press. Subsequently, a brief feedback image informing the participants about the total gain was presented. If participants pressed too late or failed to press at all (too late or miss trial, respectively), they were presented with the text message “you win nothing” plus the total gain so far. To ensure participants won similar amounts of each reward (in ±2/3 of the trials), target presentation times were determined individually and adaptively: following hit trials the response deadline for that reward cue was decreased with 10 ms, following too late or miss trials it increased with 10 ms. **(B)** Reward cues for high- and low-calorie cues [(C) Participant’s choice from cola, orange juice or chocolate milk vs. water (W)] and high and low monetary cues (50 cents vs. 1 cent).

We adapted the original incentive delay task (27) to assess neural and behavioral responses to reward anticipation following monetary as well as caloric cues (Figure 2). In short, on each trial, participants were cued about which of four rewards they could win (monetary: 1 or 50 cents; caloric: a sip of water or of a high-calorie drink of their choice [orange juice, whole chocolate milk or regular cola)], typically referred to as “reward anticipation.” As soon as a white star (target) appeared on the screen, participants had to press a button with their right index finger as fast as possible. If participants responded within an individually determined time-window, they won and the reward was added to their cumulative gain as shown on the screen. Note that rewards were not actually received during scanning. Nonetheless this phase of the task is typically referred to as “reward receipt.” On average, 59.6% (SD: 10.0) of the trials were hit trials. Participants performed 4 blocks of 25 trials (a total of a 100 trials) resulting in a task duration of 20–25 min. A block contained either high/low monetary or high/low-calorie trials. Each trial type was repeated approximately 25 times (M: 24.4, SD: 2.78). Block-presentation was pseudo-randomly distributed and counterbalanced across participants (randomization scheme: ABBA or BAAB). All stimuli were presented with a digital projector at the back end of the MRI scanner bore, which was visible via a mirror mounted on the head coil around the participant’s head. Responses were made using an MRI-compatible button box.

After scanning, participants received and drank their total caloric gain. Their total monetary gain was added to their financial reimbursement. Participants received instructions for the incentive delay task before going into the scanner and were aware they would receive their gain following scanning. Before scanning, participants rated how much they *wanted* and *liked* each reward on a Visual Analogue Scale (VAS, 100 mm). To expose participants to the reward outcomes, they were provided with the actual coins, and one sip (5 mL) of water and one of the chosen drink while rating the VAS.

### 2.5. fMRI acquisition

Participants performed the incentive delay task while whole-brain functional images were acquired on a Siemens 3 T Skyra MRI scanner (Siemens Medical system, Erlangen, Germany) using a 32-channel head coil to measure blood oxygen level dependent (BOLD) contrast. A multi-echo echo-planar imaging (EPI) sequence was used to acquire 34 axial slices per functional volume in ascending direction (voxel size 3.5 × 3.5 × 3 mm; repetition time (TR) 2,070 ms; TE 9 ms, 19.25 ms, 29.5 ms, and 39.75 ms; flip angle 90°; field of view 224 mm). This is a method that uses accelerated parallel imaging to reduce image artifacts (in plane acceleration 3) and acquire images at multiple TEs following a single excitation (45). Before the acquisition of functional images, a high-resolution anatomical scan was acquired (T1-weighted MPRAGE, voxel size 1 × 1 × 1 mm, TR 2,300 ms, TE 3.03 ms, 192 sagittal slices, flip angle 8°, field of view 256 mm).

### 2.6. Analysis

#### 2.6.1. Characterization of intervention groups

Between-group comparisons of pre-intervention measures were analyzed using independent-samples *t*-tests, Fisher’s Exact Tests, or Mann–Whitney U tests using two-tailed tests in SPSS (version 23.0, Chicago, IL). The significance level was set at an alpha of *p* = 0.05.

#### 2.6.2. Behavioral outcomes

Mean latencies of the manual responses in the incentive delay task were analyzed using repeated-measures ANOVA with within-participant factors Reward (high, low), Domain (caloric, monetary), Time (pre, post session), and the between-participant factor Intervention (ME, EC). Specific effects were tested with subsequent *F*-tests. All analyses were performed using two-tailed tests in SPSS (version 23.0, Chicago, IL). The significance level was set at an alpha of *p* = 0.05, partial eta squared (η_p_^2^) was reported to indicate effect sizes in the repeated measures ANOVAs.

#### 2.6.3. Neuroimaging outcomes

Data were pre-processed and analyzed using FSL version 5.0.11^2^ and SPM8.^3^

##### 2.6.3.1. Selection of optimal main task effects

To decide on the optimal approach for pre-processing for analysis of the mesoblimbic regions of interest, we performed three approaches, which differed in how motion-related noise was accounted for. The final approach was determined based on the strength of the main task effect (i.e., the *t*-value of the high minus low reward anticipation contrast) independent of training, i.e., across all participants and sessions. For the first approach, we accounted for motion-related noise by adding 12 rigid-body transformation parameters (three translations and rotations, and their linear derivatives) obtained during realignment to the first level model. As a second approach, we used non-aggressive ICA-AROMA (46) to reduce motion-induced signal variations in the fMRI data. Because ICA-AROMA takes out noise components, the 12 rigid-body transformation parameters obtained during realignment were not included in the model. For our third approach, we again used ICA-AROMA. However, rather than reducing motion-related noise in the fMRI data directly, we added the time courses of the independent components that accounted for less than 5% of task-related variance to the first level model. To achieve this, we used the components identified as motion by ICA-AROMA in a multiple regression analysis with the task regressors as predictors and the motion-related time courses as dependent variables. From this analysis, the adjusted *R*^2^ was obtained to identify how much of the total variance in a time course was captured by the task’s design. If the adjusted *R*^2^ of a component was higher than 5%, it was not included in the first level model as noise regressor. The 12 rigid-body transformation parameters obtained during realignment were also included in the model. The third approach showed the strongest main task effect (brain responses to high minus low reward cues) and was therefore used as our final pre-processing approach. In the next Section 2.6.3.2, we describe this approach in more detail.

##### 2.6.3.2. Preprocessing

The volumes for each echo time were realigned to correct for motion artifacts (estimation of the realignment parameters is done for the first echo and then copied to the other echoes). The four echo images were combined into a single MR volume based on 31 volumes acquired before the actual experiment started using an optimized echo weighting method (45). Combined functional images were slice-time corrected by realigning the time-series for each voxel temporally to acquisition of the middle slice. The images were subsequently spatially smoothed using an isotropic 6 mm full-width at half-maximum Gaussian kernel. Non-aggressive ICA-AROMA (46) was used to identify motion-induced signal variations in the fMRI data. Participant-specific structural and functional data were then coregistered to a standard structural or functional stereotactic space, respectively, (Montreal Neurological Institute (MNI) template). After segmentation of the structural images using a unified segmentation approach, structural images were spatially coregistered to the mean of the functional images. The resulting transformation matrix of the segmentation step was then used to normalize the anatomical and functional images into Montreal Neurological Institute space. The functional images were resampled at voxel size 2 × 2 × 2 mm.

##### 2.6.3.3. Subject-level statistics

Statistical analyses of fMRI data at the individual participant (first) level were performed using an event-related approach and included 13 regressors of interest: four regressors for cue presentation (high- and low-calorie cues, high and low monetary cues), one regressor for target presentation, four outcome regressors for hits (high- and low-calorie hits, high and low monetary hits), and four outcome regressors for trials on which participants responded too late (high- and low- calorie too late, high and low monetary too late). If participants failed to respond on a trial (i.e., a miss), the trial was excluded from analyses. Onsets of the regressors were modeled as a stick function (duration = 0 s) convolved with a canonical hemodynamic response function (47). Furthermore, we only added time courses of the independent noise components that accounted for less than 5% of task-related variance to the first level model as regressors of non-interest. Note that the number of these regressors varied per subject and session. In addition, 12 rigid-body parameters, a constant term, and two regressors that reflected signal variation in white matter and cerebrospinal fluid regions were included as regressors of non-interest. High pass filtering (128 s) was applied to the time series of the functional images to remove low-frequency drifts and correction for serial correlations was done using an autoregressive AR(1) model.

##### 2.6.3.4. Group-level statistics

**Figure 3:**
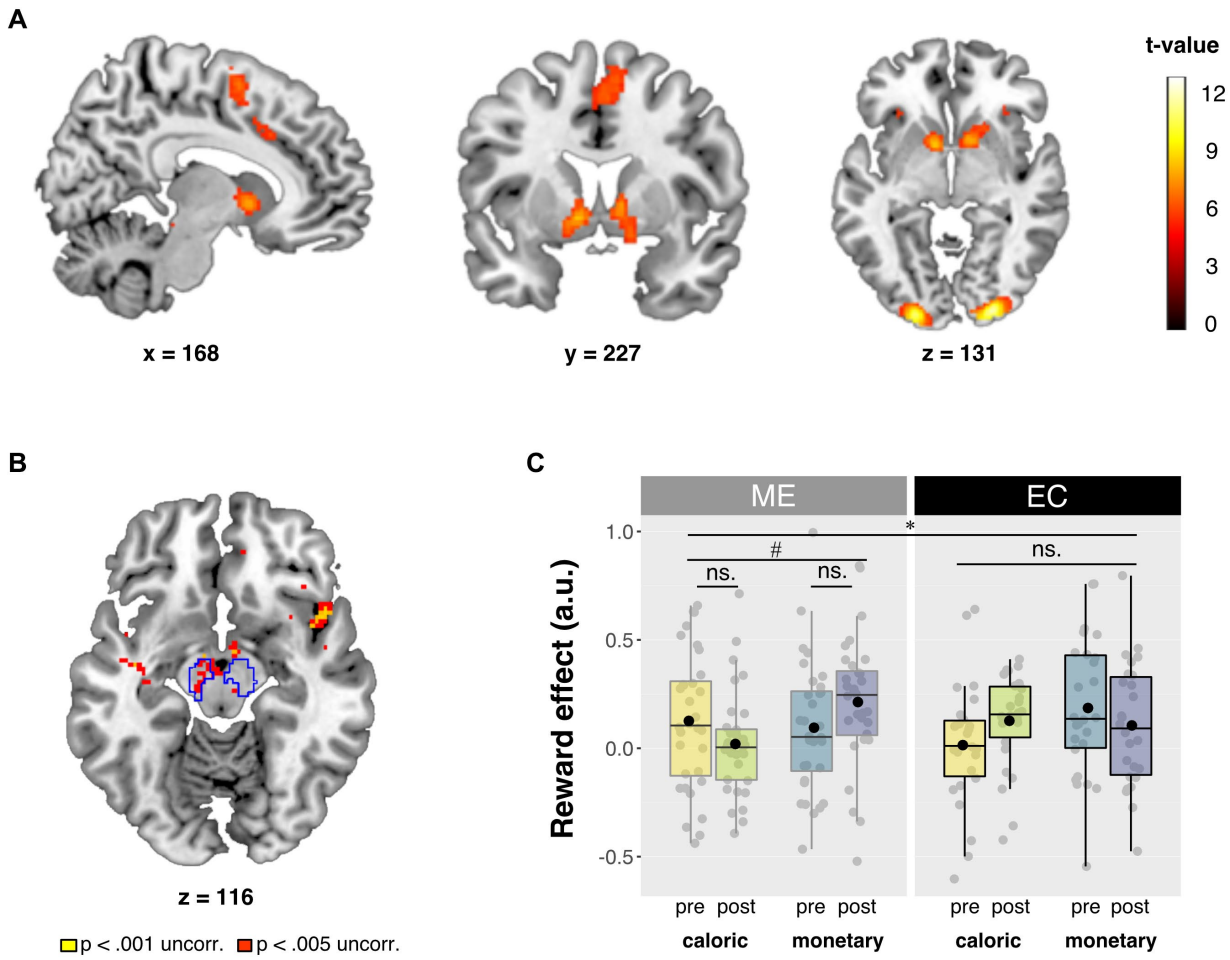
Summary of neuroimaging results. **(A)** Main effect of reward. Contrast of high vs. low reward cue trials (high > low). Full brain statistical parametric maps were thresholded at *p* < 0.05 (FWE-corrected, peak-level). **(B)** Axial slice of whole brain interaction effect of Domain × Time × Intervention for the Reward contrast (high > low). Statistical parametric maps were thresholded at *p* < 0.001 (yellow) and *p* < 0.005 (red) uncorrected for visualization purposes. Outlined regions are corrected for multiple comparisons within our small search volume, at peak pFWE < 0.05. **(C)** Betas from the bilateral probabilistic midbrain ROI [outlined in blue in panel **(B)**]. Post-minus pre-intervention mean betas based on the high minus low reward contrast are presented for each domain (caloric, monetary) and for each intervention group (ME, EC) in arbitrary units (a.u.). Box plots show the median and interquartile range, with the black dot denoting the mean. All statistical parametric maps are overlaid onto a T1-weighted canonical image. Slice coordinates are defined in MNI152 space and images are shown in neurological convention (left = left). *p < 0.025 (Bonferroni corrected for two ROIs) and ^#^*p* < 0.05.

We ran two general linear models (GLMs) at the second level: one for reward anticipation with high minus low reward cue contrast images, and one for reward receipt with hit minus too late contrast images. Analysis of variance (ANOVA) was performed in a full-factorial design, with between-subject factor Intervention and within-subject factors Time and Domain, resulting in 8 cells. Effects were considered statistically significant when reaching a threshold of *p* < 0.05, family wise error (FWE) corrected for multiple comparisons at the peak level, whole brain or in the *a priori* defined regions of interest (see below). We report whole-brain and small volume corrected (pFWE < 0.05) effects, and show the statistical maps at *p* < 0.001 and *p* < 0.005 uncorrected thresholds in Figure 3 for exploratory purposes.

##### 2.6.3.5. ROI analyses

To further investigate the effects of intervention on reward anticipation and receipt, region-of-interest (ROI) analyses were performed using *a priori* defined ROIs for midbrain and striatum. ROIs were anatomically defined based on a high-resolution probabilistic *in vivo* atlas that included midbrain and striatal nuclei (48): bilateral substantia nigra (atlas: region 7), and ventral tegmental area (region 11) for *midbrain*, and bilateral caudate nucleus (region 2), nucleus accumbens (region 3) and putamen (region 1) for *striatum* at 100% overlap. Probabilistically weighted mean beta weights were extracted from all voxels in both ROIs separately using MarsBar (49). The probabilistically weighted averaged beta-weights were analyzed per region using ANOVA with the same factors as in the whole-brain analyses. As two ROIs were tested (striatum and midbrain), effects for each total region were considered significant when reaching a threshold of *p* < 0.025 (Bonferroni corrected for multiple comparisons). *Post hoc*, the same effects were tested in the striatal sub-regions (bilateral caudate nucleus, nucleus accumbens, and putamen) because striatal sub-regions have been associated with distinct neurocognitive mechanisms.

#### 2.6.4. Anthropometric, self-reported and neuropsychological outcomes

Effects of training on anthropometric, neuropsychological and self-report measurements were analyzed using two-tailed repeated-measures ANOVA with Time (pre, post) as within-participant factor and Intervention (ME, EC) as between-participant factor. In case of violation of the assumption of sphericity as indicated by Mauchly’s test, the Huyhn-Feldt correction was used to adjust the degrees of freedom accordingly (see Section 3).

To assess the longevity of the anthropometric and self-report measures that exhibited a significant Time x Intervention interaction, we ran *post hoc* ANOVAs adding the one-year follow-up data as a third level in factor Time for BMI, waist, DHD-FFQ, and FBQ knowledge. One-year follow-up data was available of 26 participants in the ME group and 21 participants in the EC group. In case of violation of the assumption of sphericity as indicated by Mauchly’s test, the Huyhn-Feldt correction was used to adjust the degrees of freedom accordingly (see Section 3). Planned *post hoc* comparisons were performed to statistically compare follow-up data to data from both the pre- and post-test sessions separately. All analyses were performed using two-tailed tests in SPSS (version 23.0, Chicago, IL). The significance level was set at an alpha of *p* = 0.05, partial eta squared (η_p_^2^) was reported to indicate effect sizes in the repeated measures ANOVAs.

## 3. Results

### 3.1. Characterization of intervention groups

The results reported in this study are based on data from 58 healthy, right-handed participants (48 women; mean age: 31.6, SD: 11.0, range: 19–52 years; mean body mass index (BMI): 26.0, SD: 3.68, range: 19.7–34.7 kg/m2). We first confirmed that the mindful eating (ME) and educational cooking (EC) groups were well matched in terms of the minimization factors age, gender, body mass index (BMI) and experience with meditation and yoga (Table 1). Note that the average education level of the ME group was numerically, but not significantly higher than that of the EC group (*p* = 0.053). *Post hoc* correlation analyses revealed no correlations between educational level and the neural effects described below and is therefore unlikely to drive these effects. The total time participants spent on the intervention, and the number of sessions participants attended did not differ significantly between the two groups (Table 1).

### 3.2. Behavioral outcomes

As a primary objective, we assessed the effects of the intervention on reward anticipation during the incentive delay task. We start with the behavioral responses during the task (Table 2). Across sessions and intervention groups, participants responded faster on high than on low reward trials [main Reward: *F* (1, 56)=25.0, *p* < 0.001, η_p_^2^ = 0.309], thus revealing a reward benefit (Table 2). In addition, participants across sessions and intervention groups responded faster to monetary relative to caloric reward cues [main Domain: *F* (1, 56) = 17.4, *p* < 0.001, η_p_^2^ = 0.237]. We observed a reward benefit for both caloric [*F* (1, 56) = 4.5, *p* = 0.038, η_p_^2^ = 0.074] and monetary trials [*F* (1, 56) = 25.6, *p* < 0.001, η_p_^2^ = 0.314], which was, however, larger in the monetary trials [Reward × Domain interaction: *F* (1, 56) = 9.0, *p* = 0.004, η_p_^2^ = 0.139]. Participants responded faster on post- relative to pre-intervention test sessions [pre: 310.66 (SD: 21.3), post: 304.60 ms (SD: 20.8); main Time: *F* (1, 56) = 4.4, *p* < 0.041, η_p_^2^ = 0.072]. However, there was no evidence for effects of intervention type [4-way interaction between Intervention × Time × Reward × Domain [*F* (1, 56)<1], indicating that the speeding of responding on the second vs. the first session was not qualified by reward magnitude, reward type or intervention type.

**Table 2:** Task-related outcomes pre- and post-training, for each group separately, and Time (pre, post) × Intervention (ME, EC) statistics (ME, mindful eating; EC, educational cooking).

	Mindful eating (ME)				Educational cooking (EC)				*p*	Test-statistic^a^	Effect size^b^
	Pre		Post		Pre		Post				
**Primary outcome measure: response times on the incentive delay task**											
**Response times per reward type**											
Low caloric	313.7	±41.0	312.4	±33.8	322.5	±51.6	312.6	±43.8	0.319	1.0	0.018
High caloric	303.4	±33.8	299.1	±31.5	322.2	±50.0	311.8	±48.4	0.471	<1	0.009
Low monetary	313.0	±47.0	311.2	±44.2	317.4	±44.8	313.3	±49.6	0.834	<1	0.001
High monetary	294.7	±26.2	285.1	±32.5	302.3	±41.5	293.9	±43.0	0.874	<1	<0.001
**Exploratory outcome measures (manipulation check): visual analogue scales**											
**Wanting per reward type**											
Low caloric	4.5	±2.8	4.6	±2.8	4.5	±3.1	4.6	±2.8	0.987	<1	<0.001
High caloric	6.3	±2.0	5.8	±2.4	5.4	±3.0	5.6	±2.4	0.330	<1	0.017
Low monetary	1.9	±2.4	1.5	±2.0	2.2	±2.5	2.4	±2.6	0.318	1.0	0.018
High monetary	5.2	±2.8	5.4	±2.7	5.0	±3.2	5.4	±2.4	0.840	<1	0.001
**Liking per reward type**											
Low caloric	6.4	±2.3	6.1	±2.2	6.2	±2.7	6.6	±2.2	0.187	1.8	0.031
High caloric	7.2	±1.6	6.7	±2.1	6.8	±2.9	6.4	±2.7	0.783	<1	0.001
Low monetary	2.2	±2.4	2.2	±2.2	2.8	±2.4	2.8	±2.3	0.967	<1	<0.001
High monetary	5.1	±2.5	5.2	±2.4	4.4	±2.7	5.3	±2.2	0.143	2.2	0.038
Hunger^c^	5.9	±2.6	5.9	±2.7	5.9	±3.0	5.6	±2.9	0.835	<1	0.001
Thirst^c^	5.7	±2.6	5.9	±2.8	6.0	±2.4	5.5	±2.4	0.273	1.2	0.023
Satiety^c^	2.3	±2.1	2.1	±0.9	1.9	±1.1	2.1	±1.2	0.345	<1	0.017

If not otherwise stated, values denote mean ± SD.

a The reported test-statistic is the *F*-value (degrees of freedom: 1, 56).

b The reported effect size is the partial eta squared (η_p_^2^).

c Hunger, Thirst, Satiety: *N* = 55 (*N*_ME_ = 29, *N*_EC_ = 26; degrees of freedom: 1, 53).

There were also no effects of the intervention on any other behavioral task-related measures that we included to control for potentially unexpected group-differences in wanting and liking of the included rewards, or hunger, thirst, and satiety VAS ratings during the task [no Time x Intervention interactions (Table 2)].

An additional main effect of Time (pre vs. post) was only observed for response times on high reward trials, with faster responses following the interventions. No main effects of Intervention were found (Supplementary Table S1).

### 3.3. Neuroimaging outcomes

#### 3.3.1. Reward anticipation

Before assessing the intervention effects on the neural responses during reward anticipation (primary outcome), we identified brain regions that responded to reward anticipation across sessions and intervention groups (main effect of Reward condition: high>low). At our whole-brain corrected threshold (FWE < 0.05, peak-level), this contrast yielded significant responses in striatum (right caudate nucleus, right nucleus accumbens, right putamen, and left pallidum) and two right midbrain regions, as well as in occipital, motor and frontal regions (Figure 3A). Note that the optimal preprocessing pipeline was selected based on maximal main effects of reward anticipation (see Section 2.6.3.1), so no inference can be made on the magnitude of these main effects. Reward anticipation differed in mostly posterior regions for monetary vs. caloric reward cues (i.e., interaction of Domain × Reward), independent of sessions and intervention groups. For all contrasts, see Table 3.

**Table 3:** Reward anticipation.

Label	Side (left/right)	MNI-coordinates x, y, z (mm)			Size (number of voxels)	*pFWE* (peak-level)	*t-*value^a^ (peak)
**Main effect of reward: high > low** ^**b**^							
Inferior occipital lobe	R	24	−94	−4	591	<0.001	11.37
		40	−82	−14		<0.001	8.82
		34	−86	−8		<0.001	7.64
Inferior occipital lobe	L	−22	−96	−4	591	<0.001	10.43
Lingual gyrus	L	−34	−88	−14		<0.001	7.83
Pallidum	L	−10	6	−4	145	<0.001	7.99
Caudate nucleus	R	12	12	−2	267	<0.001	7.88
Nucleus accumbens	R	14	6	−12		<0.001	6.32
Putamen	R	20	18	−4	2,134	<0.001	6.05
Supplementary motor area	R	0	2	54	323	<0.001	6.87
		8	4	60		<0.001	6.60
		10	−2	66		<0.001	5.93
Insula	L	−32	26	−2	18	<0.001	6.11
Cingulate gyrus, mid part	L	−6	14	36	9	0.001	5.86
Cingulate gyrus, mid part	R	8	20	34	28	0.002	5.82
		8	12	42		0.012	5.42
Midbrain	R	10	−26	−12	3	0.006	5.58
Superior frontal gyrus	R	18	0	58	1	0.011	5.45
Inferior frontal gyrus, orbital	R	32	30	−4	7	0.027	5.26
Midbrain	R	8	−30	−12	1	0.048	5.12
**Main effect of reward: low > high reward** ^**b**^							
Superior temporal gyrus	R	62	−26	8	71	<0.001	6.87
Middle occipital lobe	L	−40	−78	4	45	<0.001	6.32
Angular gyrus	R	52	−58	28	52	0.001	6.02
		54	−62	36		0.012	5.44
Superior frontal gyrus	R	26	18	44	9	0.001	5.87
Precuneus	R	10	−50	42	19	0.002	5.81
Superior frontal gyrus	R	22	28	56	58	0.002	5.79
Inferior frontal gyrus, triangular	L	−46	38	14	3	0.003	5.69
Precuneus	R	8	−60	46	4	0.004	5.64
Inferior parietal gyrus	L	−44	−40	42	2	0.006	5.56
Insula	R	38	−14	18	2	0.031	5.22
Inferior parietal gyrus	L	−46	−42	46	1	0.033	5.21
Lingual gyrus	L	−28	−58	−4	1	0.046	5.13
**Interaction effect of Reward × Domain: caloric (high > low reward) > monetary (high > low reward)** ^**b**^							
Inferior occipital lobe	L	−48	−70	−4	595	<0.001	10.47
Fusiform gyrus	L	−38	−54	−12		<0.001	6.17
Inferior temporal gyrus	R	50	−68	−6	251	<0.001	8.41
Middle temporal gyrus	R	44	−62	−2		<0.001	7.06
Inferior temporal gyrus	R	58	−60	−10		0.005	5.60
Middle occipital lobe	L	−26	−76	28	92	<0.001	6.69
Inferior temporal gyrus	R	50	−54	−18	27	0.001	5.97
Inferior frontal gyrus, opercular	L	−46	6	28	24	0.001	5.91
Inferior parietal gyrus	L	−42	−40	42	24	0.002	5.79
Fusiform gyrus	L	−28	−58	−12	14	0.008	5.51
Middle occipital lobe	R	34	−64	36	20	0.010	5.47
Inferior parietal gyrus	L	−36	−40	36	2	0.018	5.35
Precentral gyrus	L	−38	2	30	1	0.018	5.35
Fusiform gyrus	L	−46	−60	−18	5	0.018	5.34
Inferior frontal gyrus, triangular	L	−42	34	12	2	0.020	5.33
Lingual gyrus	L	−24	−54	−10	1	0.023	5.29
Precentral gyrus	L	−42	2	30	1	0.039	5.17
Middle occipital lobe	R	32	−76	30	1	0.049	5.11
**Interaction effect of Reward × Domain: monetary (high > low reward) > caloric (high > low reward)** ^**b**^							
Inferior occipital lobe	L	−22	−96	−4	1,104	<0.001	20.14
Lingual gyrus	R	24	−92	−8	995	<0.001	18.47
**Interaction effect: Reward × Domain × Time × Intervention** ^**c**^ **(primary objective)**							
Midbrain	L	−10	−18	−10	1	0.131	3.19
Putamen	R	20	22	−2	12	0.250	3.83
Caudate nucleus	R	12	12	2	23	0.369	3.69
Caudate nucleus	R	12	8	−6		0.369	3.68
Pallidum	R	10	8	0		0.619	3.42
Caudate nucleus	R	16	14	4		0.769	3.27

Summary of brain regions exhibiting main effects of reward, domain and/or interactions with domain, intervention, and time. N.B., the preprocessing pipeline was selected based on maximal main effects of reward anticipation.

a Degrees of freedom: 1, 224.

b *p* < 0.05, whole-brain family wise error (FWE) corrected.

c pFWE value for the smaller midbrain and striatum search volumes.

We were primarily interested in the effects of mindful eating on reward anticipation in our *a priori* defined, anatomical region-of-interest (ROI): the striatum. We explored the same effects in an anatomical midbrain ROI. First, we explored these effects using our probabilistic ROIs as small search volumes. We found five peaks for the Reward × Domain × Time × Intervention interaction in the striatum (three regions in caudate nucleus, one in putamen, and one in pallidum), as well as one peak in the midbrain. However, these peaks were not significant when correcting for multiple comparisons across the two search volumes (i.e., midbrain and striatum), i.e., all pFWE > 0.025 (Figure 3B).

Based on our hypotheses, we also performed ROI analyses (Figure 3C) using a bilateral probabilistic structural ROI for the striatum (primary) and the midbrain (see Section 2.6.3.6). No four-way interaction effect (Intervention × Time × Domain × Reward) was found for the striatum. *Post hoc* analyses of the separate striatal regions also showed no effect of intervention [Intervention × Time × Domain × Reward: putamen: *F* (1, 56) < 1, *p* = 0.385, η_p_^2^ = 0.014, caudate nucleus: *F* (1, 56) = 1.3, *p* = 0.255, η_p_^2^ = 0.023, nucleus accumbens: *F* (1, 56) = 2.2, *p* = 0.142, η_p_^2^ = 0.038]. Interestingly, for the midbrain ROI, we did observe a significant four-way interaction in the ROI betas, and—in contrast to the observed midbrain effect in the small volume analysis mentioned above—this effect did survive correction for multiple comparisons [Intervention × Time × Domain × Reward: *F* (1, 56) = 7.9, *p* = 0.007, η_p_^2^ = 0.123, α = 0.025]. *Post hoc* analyses showed a significant relative reduction in caloric vs. monetary reward anticipation in midbrain after the mindful eating training [Time × Domain × Reward for ME: *F* (1, 31) = 4.4, *p* = 0.043, η_p_^2^ = 0.125]. This effect was not significant in the educational cooking group [Time × Domain × Reward for EC: *F* (1, 25) = 3.7, *p* = 0.065, η_p_^2^ = 0.130] and, if anything, showed the opposite effect. When further breaking down the interaction in the mindfulness group, we found no significant training effect in the caloric domain [Time × Reward: *F* (1, 31) = 2.1, *p* = 0.156, η_p_^2^ = 0.064], or in the monetary domain [Time × Reward: *F* (1, 31) = 2.8, *p* = 0.104, η_p_^2^ = 0.083] separately. This means that we can only interpret the ME effect on midbrain reward anticipation responses as a *relative* decrease for caloric vs. monetary reward (see above-mentioned significant Time × Domain × Reward effect for ME). Pre-intervention Reward differences could not explain the observed interaction in the midbrain [caloric: *t* (56) = 1.4, *p* = 0.169, cohen’s d = 0.370, monetary: *t* (56) = 1.1, *p* = 0.272, Cohen’s d = 0.292].

To explore whether the time spent on training affected anticipatory reward processing in the midbrain, we ran a *post hoc* analysis with total time spent on training for each participant as a covariate. Adding this covariate (and interaction terms) did not change the results [Reward × Domain × Time × Intervention: *F* (1, 55) = 7.4, *p* = 0.009, η_p_^2^ = 0.118]. Because BMI and waist showed effects of the intervention (see secondary Results: Anthropometric measures), we added BMI and waist as covariates to the analysis. This also did not change the results qualitatively [Reward × Domain × Time × Intervention interaction with BMI covariate: *F* (1, 55)=5.93, *p* = 0.018, η_p_^2^ = 0.097; with waist circumference covariate: *F* (1, 55) = 5.94, *p* = 0.018, η_p_^2^ = 0.097].

#### 3.3.2. Reward receipt

The intervention did not affect neural responses during the receipt of reward. Specifically, no significant main effects of Intervention or interactions with Intervention were found for BOLD responses to reward receipt in whole-brain analyses, nor in ROI analyses using *a priori* defined ROIs for striatum and midbrain. For main effects and other interaction effects of reward receipt see Table 4.

**Table 4:** Reward receipt.

Label	Side (left/right)	MNI-coordinates x, y, z (mm)			Size (number of voxels)	*pFWE* (peak-level)	*t-*value^a^ (peak)
**Main effect of receipt: hits (high > low) > too lates (high > low)** ^b^							
Nucleus accumbens	L	−14	6	−12	880	<0.001	13.61
Putamen	L	−18	10	−6		<0.001	12.41
Hippocampus	L	−16	−6	−16		<0.001	6.89
Putamen	R	18	8	−8	1,019	<0.001	12.50
		22	14	−4		<0.001	12.42
		30	−10	2		<0.001	8.63
Middle temporal gyrus	R	48	−72	0	1,371	<0.001	9.85
Inferior temporal gyrus	R	52	−54	−16		<0.001	8.91
Middle occipital lobe	R	32	−80	10		<0.001	7.40
Superior frontal gyrus, medial orbital	L	−4	50	−6	1,197	<0.001	9.63
		−6	42	−8		<0.001	8.35
		−6	60	2		<0.001	7.87
Superior frontal gyrus	L	−20	30	52	794	<0.001	9.38
Middle frontal gyrus	L	−22	18	46		<0.001	6.82
Superior frontal gyrus	L	−14	46	38		<0.001	6.07
Inferior temporal gyrus	L	−52	−48	−14	499	<0.001	9.26
Inferior parietal gyrus	L	−48	−40	48	1,530	<0.001	8.86
Superior parietal gyrus	L	−30	−66	48		<0.001	8.28
Inferior parietal gyrus	L	−42	−40	40		<0.001	8.14
Inferior parietal gyrus	R	34	−48	50	1,074	<0.001	8.72
Supramarginal gyrus	R	46	−36	46		<0.001	8.69
Inferior parietal gyrus	R	40	−42	50		<0.001	7.22
Inferior frontal gyrus, triangular	L	−40	36	14	324	<0.001	8.60
Putamen	L	−30	−12	4	104	<0.001	8.19
Inferior frontal gyrus, triangular	L	−36	36	12	142	<0.001	7.12
Inferior frontal gyrus, orbital	L	−26	30	−18		<0.001	6.70
Caudate nucleus	L	−20	−8	26	92	<0.001	7.01
		−20	−16	30		<0.001	6.10
Inferior frontal gyrus, opercular	L	−44	6	26	51	<0.001	6.41
Superior frontal gyrus	R	22	30	48	28	<0.001	6.26
Caudate nucleus	R	18	−8	26	43	0.001	6.03
		20	6	20		0.005	5.62
Inferior frontal gyrus, orbital	R	28	36	−14	16	0.001	5.97
Cingulate gyrus, mid part	R	4	−38	34	27	0.001	5.85
Middle occipital lobe	L	−24	−92	8	48	0.002	5.77
		−32	−88	10		0.007	5.53
Precentral gyrus	R	48	4	28	31	0.002	5.76
Inferior occipital lobe	L	−48	−74	−2	12	0.002	5.74
Middle occipital lobe	L	−26	−84	20	14	0.002	5.67
Precuneus	R	2	−62	24	23	0.004	5.66
Paracentral lobule	L	−2	−26	60	28	0.004	5.63
	R	8	−24	62		0.004	5.45
Hippocampus	L	−32	−34	−6	1	0.007	5.55
Inferior parietal gyrus	L	−26	−50	44	1	0.035	5.19
Middle temporal gyrus	L	−62	−10	−22	1	0.045	5.14
**Main effect of receipt: too lates (high > low reward) > hits (high > low reward)** ^b^							
Middle temporal gyrus	R	48	−26	−6	1,877	<0.001	13.37
		48	−36	0		<0.001	10.35
Supramarginal gyrus	R	60	−42	36		<0.001	9.06
Inferior frontal gyrus, orbital	R	48	22	−4	956	<0.001	9.42
Inferior frontal gyrus, triangular	R	54	22	4		<0.001	8.04
		44	22	8		<0.001	7.15
Supramarginal gyrus	L	−62	−44	26	342	<0.001	8.91
Supplementary motor area	R	6	24	62	1,584	<0.001	8.58
		8	14	66		<0.001	8.45
Superior frontal gyrus, medial	R	4	34	54		<0.001	7.70
Middle temporal gyrus	L	−50	−28	−4	437	<0.001	8.33
		−50	−48	8		<0.001	6.95
Thalamus	L	−8	−16	8	355	<0.001	7.51
Thalamus	R	10	−16	10		<0.001	7.35
		8	−8	6		<0.001	7.02
Middle frontal gyrus	R	30	50	24	274	<0.001	7.50
Middle temporal gyrus	L	−56	2	−14	626	<0.001	7.29
Insula	L	−34	22	−8		<0.001	7.14
		−36	20	8		<0.001	6.80
Postcentral gyrus	L	−40	−22	50	320	<0.001	7.04
		−48	−20	46		<0.001	6.30
		−42	−24	38		0.033	5.21
Middle frontal gyrus	L	−26	48	24	90	<0.001	6.89
		−24	38	20		0.013	5.41
Caudate nucleus	R	12	2	14	20	<0.001	6.24
Cingulate gyrus, mid part	R	6	−18	36	15	<0.001	6.05
Middle temporal pole	R	52	8	−22	32	0.001	5.92
Insula	R	40	0	−14	13	0.002	5.80
Caudate nucleus	L	−8	8	6	4	0.003	5.71
Cerebellum	R	22	−50	−22	7	0.004	5.66
Rolandic operculum	L	−40	−20	18	9	0.009	5.48
Superior temporal gyrus	L	−40	−4	−14	2	0.016	5.37
Middle temporal gyrus	R	50	2	−22	1	0.038	5.17
Temporal pole: superior temporal gyrus	R	50	16	−18	1	0.044	5.14
Midbrain	R	6	−24	−6	1	0.046	5.13
Calcarine fissure	R	8	−80	12	1	0.047	5.13
**Interaction effect of Domain × Reward: caloric [hits (high > low reward) > toolates (high > low reward)] > monetary [hits (high > low reward) > toolates (high > low reward)]** ^b^							
Lingual gyrus	R	18	−86	−4	195	<0.001	8.96
Calcarine fissure	L	−10	−90	−4	63	0.001	6.04
Cerebellum	R	16	−80	−16	5	0.005	5.59
**Interaction effect of Intervention × Domain × Reward** ^b^							
Putamen	R	30	−14	8	1	0.015	5.38
**Interaction effect of Domain × Receipt × Reward: monetary [hits (high > low reward) > toolates (high > low reward)] > caloric [hits (high > low reward) > toolates (high > low reward)]** ^b^							
Lingual	R	18	−84	−4	116	<0.001	7.02
Lingual	L	−14	−88	−4	1	0.023	5.01

Summary of brain regions exhibiting main effects of reward, domain and/or interactions with domain, training, and time.

a Degrees of freedom: 1,224.

b *p* < 0.05, whole-brain FWE corrected.

### 3.4. Anthropometric, self-reported and neuropsychological outcomes

As a secondary outcome, we analyzed the effects of the two interventions on the anthropometric measures. Although we initially intended to assess only BMI and waist-to-hip ratio (WHR), we have added the analysis of waist circumference as an additional exploratory measure to assess abdominal obesity (50). Changes in abdominal obesity as measured with WHR may be masked because of the relative nature of the measure [i.e., if the interventions affect waist and hip circumference similarly, especially in women (51)]. The interventions had differential effects on the anthropometric measures as indicated by a significant Time x Intervention interaction. Specifically, the active control, EC, intervention resulted in both decreased BMI and waist circumference [main Time: BMI: *F* (1, 25) = 6.2, *p* = 0.020, η_p_^2^ = 0.198; waist circumference: *F* (1, 25) = 17.9, *p* < 0.001, η_p_^2^ = 0.418], whereas the ME intervention did not affect either of them [main Time: BMI: *F* (1, 31) < 1, *p* = 0.648, η_p_^2^ = 0.007; waist circumference: *F* (1, 31) < 1, *p* = 0.504, η_p_^2^ = 0.015]. Waist-to-hip ratio was not affected by either of the interventions [Time × Intervention: *F* (1, 56) < 1, *p* = 0.379, η_p_^2^ = 0.014]. For all interactions see Table 5. Main effects of Time were also observed for waist circumference and for WHR, but not for BMI. No main effects of Intervention were observed for these measures (Supplementary Table S2).

**Table 5:** Secondary anthropometric, self-reported eating behavior, and neuropsychological outcomes.

	Mindful eating (ME)				Educational cooking (EC)				*p*	Test-statistic^a^	Effect size^b^
	Pre		Post		Pre		Post				
**Anthropometric outcomes**											
BMI (kg/m^2^)	26.6	±4.1	26.6	±4.2	25.5	±3.4	25.2	±3.5	0.023	5.5	0.089
WHR	0.85	±0.06	0.84	±0.07	0.85	±0.06	0.84	±0.07	0.379	<1	0.014
Waist (cm)	89.6	±12.8	89.3	±13.2	86.5	±11.7	84.4	±11.7	0.026	5.2	0.085
**Self-report eating behavior outcomes**											
DHD-FFQ	52.2	±10.4	54.2	±10.0	51.6	±12.0	59.5	±10.8	0.036	4.6	0.076
FBQ	64.0	±7.0	62.8	±5.6	62.1	±4.8	62.7	±6.3	0.264	1.3	0.022
Knowledge	15.6	±1.5	15.8	±1.3	14.9	±1.5	16.7	±0.8	<0.001	19.6	0.259
Temptation	15.0	±3.2	14.4	±3.3	14.8	±3.3	14.5	±4.0	0.729	<1	0.002
DEBQ											
Restraint	2.8	±0.6	2.9	±0.6	2.9	±0.7	2.9	±0.6	0.814	<1	0.001
Emotional	2.8	±0.8	2.8	±0.8	2.8	±0.7	2.7	±0.9	0.728	<1	0.002
External	3.2	±0.4	3.2	±0.5	3.4	±0.5	3.1	±0.5	0.120	2.5	0.043
**Other self-report and neuropsychological outcomes**											
FFMQ-SF^c^	78.1	±7.7	76.8	±7.4	76.5	±8.6	75.7	±7.9	0.671	<1	0.003
TCQ^d^	30.0	±7.4	27.8	±8.4	32.7	±4.8	32.8	±8.1	0.215	1.6	0.029
PANAS											
Positive affect	31.8	±6.5	30.0	±6.1	31.4	±4.8	29.8	±5.1	0.772	<1	0.002
Negative affect	12.7	±2.8	13.9	±4.3	12.7	±2.6	13.4	±3.6	0.602	<1	0.005
BIS-BAS											
BIS	20.8	±3.3	20.3	±3.2	19.8	±3.3	19.6	±3.3	0.671	<1	0.003
BAS	41.5	±3.3	42.3	±4.0	43.2	±4.1	42.7	±4.1	0.101	2.8	0.047
HADS											
Anxiety	4.4	±2.4	6.0	±2.5	4.8	±2.5	6.2	±3.9	0.902	<1	<0.001
Depression	2.6	±2.4	2.8	±2.4	2.4	±2.3	2.7	±2.6	0.864	<1	0.001
FTND (smoking score)	0.19	±1.1	0.19	±1.1	0.04	±0.2	0.04	±0.2	1.000	416^e^	<0.001^f^
BIS-11	62.0	±9.3	62.1	±9.0	64.5	±8.7	63.7	±8.3	0.492	<1	0.008
Kirby	0.013	±0.023	0.015	±0.023	0.020	±0.045	0.011	±0.017	0.094	2.9	0.049
Digit span^g^	15.6	±3.5	15.2	±3.6	14.1	±3.5	13.5	±3.7	0.689	<1	0.003

Means and standard deviations, pre- and post-training, for each group (ME, mindful eating; EC, educational cooking) separately, and Time (pre, post) × Intervention (ME, EC) statistics.

If not otherwise stated, values denote mean ± SD.

BMI, Body Mass Index; WHR, waist-to-hip ratio; DHD-FFQ, Dutch Healthy Diet Food Frequency Questionnaire; FBQ, Food Behavior Questionnaire, a shortened version; DEBQ, Dutch Eating Behaviour Questionnaire; FFMQ-SF, Five Facet Mindfulness Questionnaire – Short Form; TCQ, Treatment Credibility Questionnaire; PANAS, Positive And Negative Affect Scale; BIS-BAS, Behavioral Inhibition System – Behavioral Approach System questionnaire; HADS, Hospital Anxiety and Depression Scale; FTND, Fagerstrom Test for Nicotine Dependence; BIS-11, Barratt Impulsiveness Scale-11; Kirby: delayed reward discounting questionnaire.

a If not otherwise stated, the reported test-statistic is the *F*-value (degrees of freedom: 1, 56).

b If not otherwise stated, the reported effect size is the partial eta squared (η_p_^2^).

c FFMQ-SF: *N* = 48 (*N*_ME_ = 22, *N*_EC_ = 26; degrees of freedom: 1, 46).

d TCQ: *N* = 55 (*N*_ME_ = 29, *N*_EC_ = 26; degrees of freedom: 1, 53).

e Mann-Whitney U.

f r, effect size for Mann Whitney U-test [*z*-value divided by the total sample size (58)].

g The total score of the digit span is reported.

As another secondary outcome, we assessed intervention effects on eating-related self-reported measures. We found that EC participants reported closer compliance to the Dutch food-based guidelines for healthy eating [main Time: *F* (1, 25) = 12.8, *p* = 0.001, η_p_^2^ = 0.339] than ME participants following their intervention [main Time: *F* (1, 31) = 1.4, *p* = 0.244, η_p_^2^ = 0.044], as substantiated by a significant Time × Intervention interaction for DHD-FFQ scores. EC participants also showed a significant increase in knowledge on healthy eating following the intervention [main Time: *F* (1, 25) = 48.8, *p* < 0.001, η_p_^2^ = 0.661], whereas ME participants did not [main Time: *F* (1, 31) < 1, *p* = 0.394, η_p_^2^ = 0.024], as evidenced by a significant Time × Intervention interaction for FBQ scores. For both these measures, we also observed a main effect of Time, but not Intervention (Supplementary Table S2). The other sub-scale of the FBQ (temptation) did not show any differential intervention effects; neither did any of the sub-scales of the DEBQ (restraint, emotional, and external eating). For all interactions see Table 5. Analysis of the other self-reported and neuropsychological measurements—including those related to the intervention (FFMQ-SF, TCQ), affect (PANAS, BIS-BAS, HADS), impulsivity (FTND, BIS-11, Kirby), and working memory (digit span) revealed no significant interactions between Time and Intervention (Table 5). A main effect of Time was observed for positive affect, as assessed on the PANAS, and for people’s anxiety score on the HADS. Across groups, positive affect was lower following the interventions, whereas the anxiety score had increased (Supplementary Table S2).

**Figure 4:**
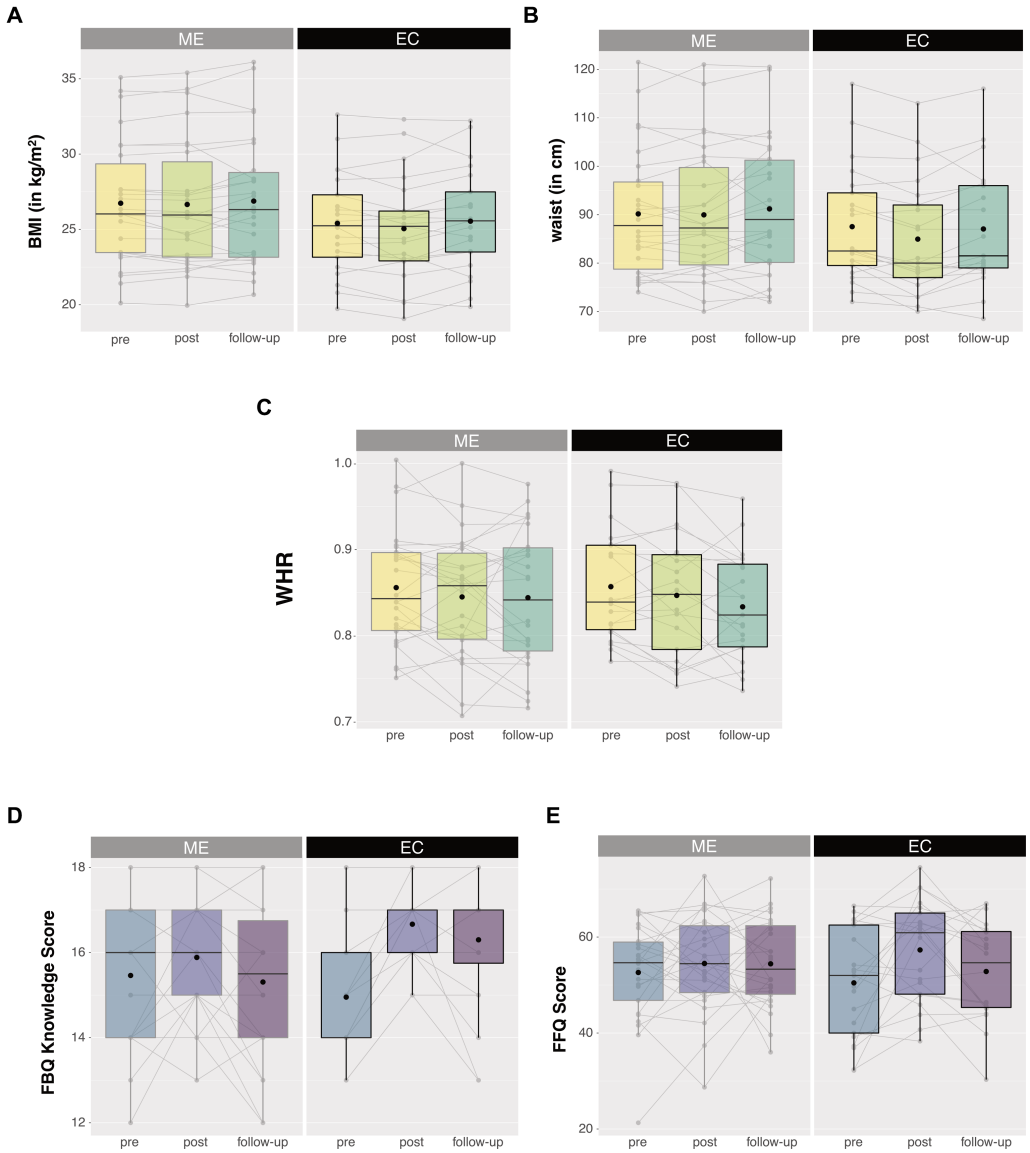
Anthropometric (upper panels) and eating-related self-report measures (lower panels) 1 year after the intervention. No long-lasting intervention effects were observed for **(A)** BMI, **(B)** waist circumference, **(C)** waist-to-hip ratio (WHR), and **(E)** compliance to the Dutch guidelines for healthy diet (DHD-FFQ). Only knowledge on healthy eating **(D)** remained high following the educational cooking (EC) intervention. No intervention effects were observed for the mindful eating (ME) group. Box plots show the median and interquartile range, with the black dot denoting the mean. Note that the medians of the EC group in figure **(D)** do not fall in the interquartile range. Individual data points at the different test sessions are connected for illustrative purpose. **Asterisks denote a significant Time × Intervention interaction with *p* < 0.01.

To establish whether the observed differential intervention effects (Time × Intervention interactions) in the anthropometric and eating-related self-report measures were long-lasting, we ran *post hoc* analyses by adding the one-year follow-up data as an extra level of factor Time (pre, post, follow-up) in the ANOVAs for all participants from the reported sample that returned for the follow-up (ME: *n* = 26, EC: *n* = 20) (Figure 4). For BMI and waist circumference, degrees of freedom were corrected using Huynh-Feldt estimates of sphericity due to violation of the sphericity assumption. BMI, WHR, and waist circumference did not show any long-term intervention-related changes [Intervention × Time, BMI: *F* (1.550, 69.77) < 1, *p* = 0.468, η_p_^2^ = 0.015; WHR: *F* (2, 44) < 1, *p* = 0.589, η_p_^2^ = 0.024; waist: *F* (1.742, 78.384) = 2.213, *p* = 0.123, η_p_^2^ = 0.047]. BMI, WHR, and waist circumference changed over time irrespective of the intervention [main Time, BMI: *F* (1.550, 69.77) = 3.730, *p* = 0.039, η_p_^2^ = 0.077; WHR: *F* (2, 44) = 5.099, *p* = 0.010, η_p_^2^ = 0.188; waist: *F* (1.742, 78.384) = 4.837, *p* = 0.014, η_p_^2^ = 0.097]. Planned *post hoc* comparisons revealed that the non-significant intervention effects on the anthropometric measures—after including the follow-up time point—were caused by a lack of significant differences between pre-intervention measurements and one-year follow-up measurements for either group (all *p* > 0.1). This means that the BMI- and waist circumference-reducing effects of the active control (EC) intervention (vs. the mindful eating intervention) were no longer visible at one-year follow-up.

The EC-related increase in knowledge on healthy eating remained significant after including the one-year follow-up time point [Intervention × Time, FBQ knowledge: *F* (2, 44) = 7.4, *p* = 0.002, η_p_^2^ = 0.253], caused by a lingering increase in knowledge on healthy eating for EC participants at one-year follow-up relative to pre-intervention measurements [*F* (1, 20) = 17.06, *p* = 0.001, η_p_^2^ = 0.460]. In contrast, the effects of the active control (EC) intervention on self-reported compliance to the Dutch guidelines for healthy diet were not long lasting (Intervention × Time, DHD-FFQ: *F* (2, 43) = 2.121, *p* = 0.132, η_p_^2^ = 0.090). Similar to the anthropometric measures, planned *post-hoc* comparisons revealed that these DHD-FFQ scores were comparable between the pre-intervention and one-year follow-up measures for either group (all *p* > 0.1), meaning that the previously observed post-pre effects of the active control (EC) intervention were only short lasting.

## 4. Discussion

The primary objective of this study was to investigate the effects of an 8-week mindful eating intervention on striatal reward anticipation responses as well as response times during an incentive delay task in healthy adults who are highly motivated to change their eating habits. In addition to the striatum, we explored these effects in the midbrain—as part of the mesolimbic reward circuit with its dopaminergic projections to the striatum (4, 5)—as regions of interests (ROIs). We observed that mindful eating training significantly impacted reward anticipation in the midbrain relative to the active control intervention (i.e., educational cooking training), with relatively reduced caloric vs. monetary reward responses in this region after the intervention. This effect was small and did not reach significance in the whole-brain corrected analysis. We found no effect of the interventions in the striatum or on response times during the incentive delay task. Previous studies have shown that greater subcortical reward responses to caloric cues, particularly in striatum, are associated with obesity (52, 53), with weight gain (9), and with increased snack food intake in healthy-weight to overweight individuals (7). Despite this clear involvement of striatum in food reward anticipation and its relationship with eating behavior, we found no effects of mindful eating training on striatal BOLD responses. Below, we interpret these null results. Anthropometric measures of adiposity (i.e., secondary outcome: BMI) temporarily decreased and self-reported (knowledge of) healthy food intake (i.e., secondary outcome: eating behavior questionnaires) increased following the educational cooking intervention, but not following the mindful eating intervention.

Although not *a priori* hypothesized, the relatively reduced anticipatory responses to the caloric (i.e., high-calorie drink vs. water) compared with the monetary (50 ct. vs. 1 ct.) cues in midbrain following the mindful eating intervention may not be surprising. Dopaminergic midbrain neurons are crucial for processing predicted reward value (5, 54) and, in concert with striatum, modulate motivated behavior such as eating (55). In line with this, Small et al. (56) showed that midbrain activity, as measured with positron emission tomography (H_2_^15^O), decreased with reduced self-reported reward value of chocolate in a sample of healthy individuals consuming chocolate beyond satiety. In another study, midbrain BOLD responses to sips of palatable milkshake were found to positively correlate with subsequent *ad libitum* milkshake intake in a group of healthy-weight to moderately obese individuals (57). Moreover, overweight and obese compared with normal weight adolescents showed increased activations in midbrain during anticipation of decisions involving risk and reward (12). Furthermore, both midbrain and striatal BOLD responses to palatable food pictures were found to correlate positively with self-reported reward drive in healthy individuals (58). These (indirect) measures of motivated eating behavior are thus associated with greater mesolimbic responses when processing food reward value. The specificity of our results for midbrain, not striatum, finds resonance in a study in healthy individuals by O’Doherty et al. (59). This study found significant responses to cues predicting the receipt of a glucose solution vs. a neutral taste in midbrain only, whereas both midbrain and striatum were responsive to cues predicting the receipt of a sweet vs. an aversive salty taste. The latter contrast may be a more sensitive, given its valence contrast, than our caloric vs. water contrast, which was perhaps not sufficiently sensitive to show intervention effects in the striatum—despite showing main task effects of reward anticipation. Given the coding of predicted reward in the midbrain, we speculate that the currently observed relative effect of the mindful eating intervention on anticipatory midbrain responses to caloric vs. monetary cues suggests that mindful eating practice may be able to reduce the impact of food cues on reward processing.

Our finding that anticipatory midbrain responses were relatively reduced in the caloric vs. monetary domain is in line with a previous study showing that only a brief 50-min mindful eating workshop (vs. an educational video) reduced subsequent impulsive choice patterns for food-, but not money-related outcomes (60). However, in studies comparing meditators with non-meditating controls, meditators exhibited reduced striatal BOLD responses to primary reward prediction errors (25) as well as monetary reward anticipation (24). In the latter study, Kirk et al. (24) compared meditators to non-meditators without a baseline measurement. The observed decrease in striatal reward processing could thus be due to pre-existing between-group differences (61). Since the present study was actively controlled including pre and post measurements, the current effects can be more reliably ascribed to the mindfulness intervention. Kirk et al. (26) also performed a similar randomized actively controlled study including pre and post measurements and found that vmPFC value signals were modulated by the mindfulness intervention for both primary (juice) and secondary (monetary) rewards. These general reward effects vs. our relative caloric vs. monetary effects might be due to both the type of intervention [general MBSR in Kirk et al. (26) vs. mindful eating presently] as well as the study sample. Specifically, in our study, participants were highly motivated to change undesired eating habits and their mindfulness practice was targeted at overcoming those – including homework practices such as resisting impulsive eating behaviors. Moreover, note that we did not observe any effects of either the ME or the EC intervention on neural responses at the time of caloric or monetary reward receipt. One might have expected reductions in vmPFC BOLD responses following the mindfulness-based intervention as was reported by Kirk et al. (26) for juice delivery (i.e., reward receipt), which we did not observe in whole-brain analysis. Other important differences with the current study are that we used promised (i.e., delivered after scanning) instead of actual rewards (delivered during scanning), and our design was optimized for investigating reward anticipation, with perhaps not enough successful reward receipt trials (i.e., approximately 33% of all anticipated rewards were missed). A meta-analysis of BOLD responses in incentive delay tasks shows consistent activation of vmPFC for reward receipt, but not anticipation (62). Together, our results suggest that a targeted mindful eating—instead of general mindfulness—intervention may have more specific effects on caloric vs. monetary reward anticipation.

The question arises whether mindfulness affects midbrain responses through top-down or bottom-up processes. Current theories on mindfulness-based interventions emphasize that improvements in emotion regulation occur through increased prefrontal cortex-mediated top-down control of regions processing affect, such as the amygdala (63, 64). An alternative way to reducing incentive motivation is through extinction during mindfulness practice, akin to exposure therapy (63, 64), which would rather be a bottom-up process. Practicing mindful eating requires one to actively withhold or interrupt cue-triggered eating, a process that may lead to extinction of conditioned responses to highly caloric stimuli (63, 65, 66) as well as the formation of new memories related to those stimuli (i.e., not reacting to them). As a result, choices for high caloric foods may be further reduced (67, 68). However, incentive motivation could also be reduced through other bottom-up effects on, for example, physiological state rather than through extinction. Increased awareness of states like hunger or satiety (69) are known to modulate conditioned responses to reward-related cues (70). Future confirmatory studies are needed to verify the exploratory midbrain findings and investigate the underlying bottom-up vs. top-down mechanisms, for instance by employing tasks manipulating top-down control on food reward processes, addressing the effects of physiological state and interoception, and by employing connectivity analyses between cortical and mesolimbic regions.

The present mindful eating effects on caloric vs. monetary reward anticipation in the midbrain were not accompanied by changes in our secondary outcome measures related to real-life eating behavior, i.e., reductions in weight, waist-hip ratio or waist circumference, or changes in self-reported eating behavior. Several other studies have found that an intensive mindful eating intervention did lead to reduced measures associated with overeating such as consumption of sweets (22), binges, externally and emotionally driven eating (71) and reductions in BMI (20) in non-clinical populations, as well as number of binges in binge-eating disorder (18). On the other hand, a more recent review by Warren et al. (72) concludes that there is a lack of compelling evidence of mindfulness and mindful eating interventions leading to a reduction in weight. The current lack of mindful eating intervention-related reductions in our secondary measures of adiposity might well reflect the heterogeneity of the included sample. People differed widely in their motivation to take part in the intervention program, which was not necessarily a desire to lose weight. Also, people’s weight status ranged from normal-weight to moderately obese, with an average BMI of only slightly overweight, whereas larger mindfulness-related reductions in food intake have been seen in overweight and obese populations in previous studies (72). Moreover, the study design—including sample size—was optimized for the primary outcome measure (i.e., neural effects) and plausibly underpowered for showing these food behavior-related effects after the mindful eating intervention. We were also not able to show increased self-reported mindfulness after the intervention on the established short version of the Five Facet Mindfulness Questionnaire (33), but this questionnaire could only be employed in a sub group (in *n* = 22 of the total *n* = 32 ME vs. *n* = 26 EC). However, ineffectiveness of our mindful eating intervention is highly unlikely given the observed midbrain findings in the hypothesized direction here (although exploratory) and our previously published effects on behavioral flexibility (28). Sampling a greater and more homogeneous population in terms of BMI and people’s motivation to change their eating habits is advised for future studies to be able to confirm reduced mesolimbic reward responses and demonstrate its link with altered eating behavior following a mindful eating intervention. For now, it is unclear how mindfulness-induced reductions in midbrain responses to caloric vs. monetary reward anticipation contribute to changes in real-life eating behavior.

In contrast, we did observe beneficial effects of the educational cooking intervention on anthropometric measures of obesity and self-reported eating behavior, whereas this group did not demonstrate any intervention effects on mesolimbic reward anticipatory responses. The beneficial effects might not be surprising for this group, since the educational cooking intervention was explicitly aimed at promoting healthy food intake, with reduced intake of sugar, fats and salt as part of the homework assignments. This led to increased self-reported adherence to the Dutch healthy diet (DHD-FFQ) and short-term reductions in weight and waist circumference. Given those health benefits of the educational cooking intervention and the relatively reduced food reward anticipation responses of the mindful eating intervention, it might be fruitful to develop a combined program for therapeutic practice or for preventive strategies. Although weight control and diet interventions are often successful in producing significant weight loss on the short term, they often fail to produce long-term weight maintenance (73). This is supported by our analyses of BMI, waist circumference, and self-reported compliance to the Dutch healthy diet guidelines (DHD-FFQ) at one-year follow-up in the present study. These secondary measures returned to baseline 1 year after the educational cooking intervention, despite the fact that knowledge of healthy eating remained significantly higher compared with baseline in the educational cooking group. Previous studies investigating factors contributing to successful weight maintenance have shown that reductions in subcortical responses to food reward cues may be beneficial for prevention or treatment of obesity (7–9). We therefore speculate that a combination of the two interventions with a focus on both information and behavior might lead to longer-lasting health benefits than either intervention on its own.

Although a strength of this study, by enabling observed effects to be actually attributed to mindfulness practice (64), we note that the lack of a—likely, very subtle—effect of mindful eating, e.g., on striatal reward anticipation, might well reflect the inclusion of a well-matched active control intervention. The contrast between the impact of the mindful eating and control intervention on people is undoubtedly smaller than when a mindful eating intervention had been compared with a waitlist control group. Although the exercises and contents of the two interventions did not overlap, we cannot exclude the possibility that the cooking workshops offered in the active control intervention implicitly led to some increase in experiential awareness as was the aim of the mindful eating intervention. Furthermore, we were not able to offer participants the opportunity to take part in the other intervention program after completion of the study, which is commonly done for mindfulness studies that include a waitlist control group, and which, based on anecdotal evidence reported by the experimenters diminished some people’s motivation to complete the intervention program they had been assigned to in this study. As such, the design of our randomized, active-controlled nature of the study was probably also the reason for a high dropout rate and lower than anticipated sample size. To address differences in results of previous mindfulness or meditation studies without active control condition, future mindfulness intervention studies, especially those aimed at unraveling subtle mechanistic effects, are recommended to not only include a well-matched active control intervention but also a waitlist control group.

In conclusion, we found that an intensive mindful eating intervention reduced midbrain food, relative to monetary, reward anticipation. These results have to be confirmed in future studies, as we primarily hypothesized striatal effects, and the midbrain findings are the result of exploratory ROI analyses, which did not reach significance in the whole-brain corrected analysis. Future studies are also required to demonstrate the clinical relevance of mindfulness-mediated reductions in food anticipation for counteracting reward cue-driven overeating, particularly given that we did not observe mindfulness-related changes in anthropometric or eating behavior measures. Given the success of mindfulness-based programs in reducing symptoms of other reward-related disorders such as substance use (74, 75) and problem gambling (76), our findings of relatively specific reduced anticipatory reward responses may also be relevant for these other targets of abuse.

## Data availability statement

The datasets presented in this study can be found in online repositories. The names of the repository/repositories and accession number(s) can be found at: Mendeley Data, http://dx.doi.org/10.17632/fthcv3kns9.1.

## Ethics statement

The studies involving human participants were reviewed and approved by Commissie Mensgebonden Onderzoek regio Arnhem-Nijmegen. The patients/participants provided their written informed consent to participate in this study.

## Author contributions

EA acquired funding for the study. EA, LJ, RC, AS, and JV designed the study. LJ, ID, IL, and JW acquired and analyzed the data, supervised by EA and RC. AS and JV supervised the execution of the interventions. LJ, ID, and EA wrote the first version of the manuscript. All authors contributed to the article and approved the submitted version.

## Funding

This work was supported by a VENI grant (016.135.023) of The Netherlands Organization for Scientific Research (NWO) and an AXA Research Fund fellowship (Ref: 2011) to EA. RC was supported by the James S. McDonnell Foundation (220020328) and a VICI grant (453-14-005) of NWO.

## Conflict of interest

The authors declare that the research was conducted in the absence of any commercial or financial relationships that could be construed as a potential conflict of interest.

## Publisher’s note

All claims expressed in this article are solely those of the authors and do not necessarily represent those of their affiliated organizations, or those of the publisher, the editors and the reviewers. Any product that may be evaluated in this article, or claim that may be made by its manufacturer, is not guaranteed or endorsed by the publisher.

## Footnotes

^1^ www.voedingscentrum.nl

^2^ http://www.fmrib.ox.ac.uk/fsl/

^3^ www.fil.ion.ucl.ac.uk/spm
