# Supplementary material for: The effects of an 8-week mindful eating intervention on anticipatory reward responses in striatum and midbrain
Source: Front Nutr. 2023 Aug 11;10:1115727. doi: 10.3389/fnut.2023.1115727 (PMC10457123; doi:10.3389/fnut.2023.1115727)
Supplement: Supplementary file 1 [file Data_Sheet_1.docx]

Supplemental tables Janssen, Duif et al.

**Supplemental Table 1.** **Task-related outcomes pre- and post-training, for each group (mindful eating, ME; educational cooking, EC) separately, with statistics of the main effects of Time (pre, post) and Intervention (ME, EC).**

|  |  | | | | | |  | | | | | | | | *Main effect of Time* | | | | *Main effect of Intervention* | | |
| --- | --- | --- | --- | --- | --- | --- | --- | --- | --- | --- | --- | --- | --- | --- | --- | --- | --- | --- | --- | --- | --- |
|  | mindful eating (ME) | | | | | | educational cooking (EC) | | | | | | | | *p* | *test-statistic^a^* | *effect size^b^* | | *p* | *test-statistic^a^* | *effect size^b^* |
|  | pre | | post | | | | pre | | | | post | | | |  |  |  |  |  |  |  |
| **Primary outcome measure**: Response times on the incentive delay task | | | | | | | | | | | | | | | | | | |  |  |  |
| Response Times per reward type | | | | | | | | | | | | | | | | | | |  |  |  |
| Low caloric | 313.7 | ±41.0 | 312.4 | | ±33.8 | | 322.5 | | ±51.6 | | 312.6 | | ±43.8 | | .193 | 1.7 | .030 | | .665 | < 1 | .003 |
| High caloric | 303.4 | ±33.8 | 299.1 | | ±31.5 | | 322.2 | | ±50.0 | | 311.8 | | ±48.4 | | .079 | 3.2 | .054 | | .118 | 2.5 | .043 |
| Low monetary | 313.0 | ±47.0 | 311.2 | | ±44.2 | | 317.4 | | ±44.8 | | 313.3 | | ±49.6 | | .569 | < 1 | .006 | | .773 | < 1 | .002 |
| High monetary | 294.7 | ±26.2 | 285.1 | | ±32.5 | | 302.3 | | ±41.5 | | 293.9 | | ±43.0 | | **.015** | 6.3 | .102 | | .350 | < 1 | .016 |
| **Exploratory outcome measures (manipulation check):** Visual analogue scales | | | | | | | | | | | | | | | | | | |  |  |  |
| Wanting per reward type | | | | | | | | | | | | | | | | | | |  |  |  |
| Low caloric | 4.5 | ±2.8 | 4.6 | | ±2.8 | | 4.5 | | ±3.1 | | 4.6 | | ±2.8 | | .739 | < 1 | .002 | | .951 | < 1 | < .001 |
| High caloric | 6.3 | ±2.0 | 5.8 | | ±2.4 | | 5.4 | | ±3.0 | | 5.6 | | ±2.4 | | .685 | < 1 | .003 | | .295 | 1.1 | .020 |
| Low monetary | 1.9 | ±2.4 | 1.5 | | ±2.0 | | 2.2 | | ±2.5 | | 2.4 | | ±2.6 | | .799 | < 1 | .001 | | .277 | 1.2 | .021 |
| High monetary | 5.2 | ±2.8 | 5.4 | | ±2.7 | | 5.0 | | ±3.2 | | 5.4 | | ±2.4 | | .347 | < 1 | .016 | | .925 | < 1 | < .001 |
| Liking per reward type | | | | | | | | | | | | | | | | | | | | |  |
| Low caloric | 6.4 | ±2.3 | | 6.1 | | ±2.2 | | 6.2 | | ±2.7 | | 6.6 | | ±2.2 | .871 | < 1 | < .001 | .785 | | < 1 | .001 |
| High caloric | 7.2 | ±1.6 | | 6.7 | | ±2.1 | | 6.8 | | ±2.9 | | 6.4 | | ±2.7 | .160 | 2.0 | .035 | .543 | | < 1 | .007 |
| Low monetary | 2.2 | ±2.4 | | 2.2 | | ±2.2 | | 2.8 | | ±2.4 | | 2.8 | | ±2.3 | .877 | < 1 | < .001 | 1.2 | | .285 | .020 |
| High monetary | 5.1 | ±2.5 | | 5.2 | | ±2.4 | | 4.4 | | ±2.7 | | 5.3 | | ±2.2 | .093 | 2.9 | .050 | .656 | | 0.2 | .004 |
| Hunger ^c^ | 5.9 | ±2.6 | | 5.9 | | ±2.7 | | 5.9 | | ±3.0 | | 5.6 | | ±2.9 | .777 | < 1 | .002 | .819 | | < 1 | < .001 |
| Thirst ^c^ | 5.7 | ±2.6 | | 5.9 | | ±2.8 | | 6.0 | | ±2.4 | | 5.5 | | ±2.4 | .762 | <1 | .002 | .987 | | < 1 | < .001 |
| Satiety ^c^ | 2.3 | ±2.1 | | 2.1 | | ±0.9 | | 1.9 | | ±1.1 | | 2.1 | | ±1.2 | .983 | < 1 | < .001 | .565 | | < 1 | .006 |

If not otherwise stated, values denote mean±SD. Note that these values are identical to those shown in **Table 2** in the main text.

^a^The reported test-statistic is the F-value (degrees of freedom: 1,56)

^b^The reported effect size is the partial eta squared (η_p_^2^)

^c^Hunger, Thirst, Satiety: N = 55 (N_ME_ = 29, N_EC_ = 26; degrees of freedom: 1,53)

**Supplemental Table 2. Secondary anthropometric, self-reported eating behaviour, and neuropsychological outcomes. Means and standard deviations, pre- and post-training, for each group (mindful eating, ME; educational cooking, EC) separately, with statistics of the main effects of Time (pre, post) and Intervention (ME, EC).**

|  |  | | | | | |  | | | | | | | | *Main effect of Time* | | | *Main effect of Intervention* | | |
| --- | --- | --- | --- | --- | --- | --- | --- | --- | --- | --- | --- | --- | --- | --- | --- | --- | --- | --- | --- | --- |
|  | mindful eating (ME) | | | | | | educational cooking (EC) | | | | | | | | *p* | *test-statistic^a^* | *effect size^b^* | *p* | *test-statistic^a^* | *effect size^b^* |
|  | pre | | post | | | | pre | | | | post | | | |  |  |  |  |  |  |
| **Anthropometric outcomes** | | | | | | | | | | | | | | | | | |  |  |  |
| BMI (kg/m^2^) | 26.6 | ±4.1 | 26.6 | | ±4.2 | | 25.5 | | ±3.4 | | 25.2 | | ±3.5 | | .080 | 3.2 | .054 | .225 | 1.5 | .026 |
| WHR | 0.85 | ±0.06 | 0.84 | | ±0.07 | | 0.85 | | ±0.06 | | 0.84 | | ±0.07 | | **.027** | 5.2 | .084 | .885 | < 1 | < .001 |
| Waist (cm) | 89.6 | ±12.8 | 89.3 | | ±13.2 | | 86.5 | | ±11.7 | | 84.4 | | ±11.7 | | **.002** | 10.7 | .160 | .224 | 1.5 | .026 |
| **Self-report eating behavior outcomes** | | | | | | | | | | | | | | | | | |  |  |  |
| DHD-FFQ | 52.2 | ±10.4 | | 54.2 | | ±10.0 | | 51.6 | | ±12.0 | | 59.5 | | ±10.8 | **<.001** | 13.1 | .188 | .350 | < 1 | .016 |
| FBQ | 64.0 | ±7.0 | | 62.8 | | ±5.6 | | 62.1 | | ±4.8 | | 62.7 | | ±6.3 | .697 | < 1 | .003 | .475 | < 1 | .009 |
| Knowledge | 15.6 | ±1.5 | | 15.8 | | ±1.3 | | 14.9 | | ±1.5 | | 16.7 | | ±0.8 | **<.001** | 31.5 | .360 | .724 | < 1 | .002 |
| Temptation | 15.0 | ±3.2 | | 14.4 | | ±3.3 | | 14.8 | | ±3.3 | | 14.5 | | ±4.0 | .239 | 1.4 | .025 | .931 | < 1 | < .001 |
| DEBQ |  |  | |  | |  | |  | |  | |  | |  |  |  |  |  |  |  |
| Restraint | 2.8 | ±0.6 | | 2.9 | | ±0.6 | | 2.9 | | ±0.7 | | 2.9 | | ±0.6 | .369 | < 1 | .014 | .640 | < 1 | .004 |
| Emotional | 2.8 | ±0.8 | | 2.8 | | ±0.8 | | 2.8 | | ±0.7 | | 2.7 | | ±0.9 | .200 | 1.7 | .029 | .850 | < 1 | < .001 |
| External | 3.2 | ±0.4 | | 3.2 | | ±0.5 | | 3.4 | | ±0.5 | | 3.1 | | ±0.5 | **.002** | 10.2 | .153 | .675 | < 1 | .003 |
| **Other self-report and neuropsychological outcomes** | | | | | | | | | | | | | | | | | |  |  |  |
| FFMQ-SF^c^ | 78.1 | ±7.7 | | 76.8 | | ±7.4 | | 76.5 | | ±8.6 | | 75.7 | | ±7.9 | .168 | 2.0 | .041 | .553 | < 1 | .008 |
| TCQ^d^ | 30.0 | ±7.4 | | 27.8 | | ±8.4 | | 32.7 | | ±4.8 | | 32.8 | | ±8.1 | .236 | 1.4 | .026 | .**030** | 5.0 | .086 |
| PANAS |  |  | |  | |  | |  | |  | |  | |  |  |  |  |  |  |  |
| Positive Affect | 31.8 | ±6.5 | | 30.0 | | ±6.1 | | 31.4 | | ±4.8 | | 29.8 | | ±5.1 | **.001** | 11.2 | .167 | .853 | < 1 | < .001 |
| Negative Affect | 12.7 | ±2.8 | | 13.9 | | ±4.3 | | 12.7 | | ±2.6 | | 13.4 | | ±3.6 | .062 | 3.6 | .061 | .733 | < 1 | .002 |
| BIS-BAS |  |  | |  | |  | |  | |  | |  | |  |  |  |  |  |  |  |
| BIS | 20.8 | ±3.3 | | 20.3 | | ±3.2 | | 19.8 | | ±3.3 | | 19.6 | | ±3.3 | .173 | 1.9 | .033 | .323 | 1.0 | .017 |
| BAS | 41.5 | ±3.3 | | 42.3 | | ±4.0 | | 43.2 | | ±4.1 | | 42.7 | | ±4.1 | .736 | < 1 | .002 | .280 | 1.2 | .021 |
| HADS |  |  | |  | |  | |  | |  | |  | |  |  |  |  |  |  |  |
| Anxiety | 4.4 | ±2.4 | | 6.0 | | ±2.5 | | 4.8 | | ±2.5 | | 6.2 | | ±3.9 | **<.001** | 13.8 | .198 | .656 | < 1 | .004 |
| Depression | 2.6 | ±2.4 | | 2.8 | | ±2.4 | | 2.4 | | ±2.3 | | 2.7 | | ±2.6 | .339 | < 1 | .016 | .777 | < 1 | .001 |
| BIS-11 | 62.0 | ±9.3 | | 62.1 | | ±9.0 | | 64.5 | | ±8.7 | | 63.7 | | ±8.3 | .555 | < 1 | .006 | .377 | < 1 | .014 |
| Kirby | 0.013 | ±0.023 | | 0.015 | | ±0.023 | | 0.020 | | ±0.045 | | 0.011 | | ±0.017 | .348 | < 1 | .016 | .865 | < 1 | < .001 |
| Digit Span *^e^* | 15.6 | ±3.5 | | 15.2 | | ±3.6 | | 14.1 | | ±3.5 | | 13.5 | | ±3.7 | .104 | 2.7 | .047 | .085 | 3.1 | .052 |

If not otherwise stated, values denote mean±SD. Note that these values are identical to those shown in **Table 5** in the main text.

*Abbreviations: BMI: Body Mass Index; WHR: waist-to-hip ratio; DHD-FFQ*: Dutch Healthy Diet Food Frequency Questionnaire; *FBQ*: Food Behavior Questionnaire, a shortened version; *DEBQ*: Dutch Eating Behaviour Questionnaire; *FFMQ-SF*: Five Facet Mindfulness Questionnaire – Short Form; *TCQ*: Treatment Credibility Questionnaire; *PANAS*: Positive And Negative Affect Scale; *BIS-BAS*: Behavioral Inhibition System - Behavioral Approach System questionnaire; *HADS*: Hospital Anxiety and Depression Scale; *BIS-11*: Barratt Impulsiveness Scale-11; *Kirby*: delayed reward discounting questionnaire.

^a^If not otherwise stated, the reported test-statistic is the F-value (degrees of freedom: 1,56)

^b^If not otherwise stated, the reported effect size is the partial eta squared (η_p_^2^)

^c^FFMQ-SF: N = 48 (N_ME_ = 22, N_EC_ = 26; degrees of freedom: 1,46)

^d^TCQ: N = 55 (N_ME_ = 29, N_EC_ = 26; degrees of freedom: 1,53).

*^e^* The total score of the digit span is reported
